# Aerosol vaccination of chicken pullets with irradiated avian pathogenic *Escherichia coli* induces a local immunostimulatory effect

**DOI:** 10.3389/fimmu.2023.1185232

**Published:** 2023-05-16

**Authors:** Sina Bagheri, Taniya Mitra, Surya Paudel, Mohamed Kamal Abdelhamid, Simon Könnyü, Viskam Wijewardana, Richard Thiga Kangethe, Giovanni Cattoli, Manolis Lyrakis, Claudia Hess, Michael Hess, Dieter Liebhart

**Affiliations:** ^1^ Clinic for Poultry and Fish Medicine, Department for Farm Animals and Veterinary Public Health, University of Veterinary Medicine, Vienna, Austria; ^2^ Animal Production and Health Laboratory, Joint FAO/IAEA Centre of Nuclear Techniques in Food and Agriculture, International Atomic Energy Agency (IAEA), Vienna, Austria; ^3^ Platform for Bioinformatics and Biostatistics, Department of Biomedical Sciences, University of Veterinary Medicine, Vienna, Austria

**Keywords:** APEC, chicken, colibacillosis, immune response, cytokine, T cells, interferon gamma

## Abstract

The present study investigated the expression of cytokines and cellular changes in chickens following vaccination with irradiated avian pathogenic *Escherichia coli* (APEC) and/or challenge. Four groups of 11-week-old pullets, each consisting of 16 birds were kept separately in isolators before they were sham inoculated (N), challenged only (C), vaccinated (V) or vaccinated and challenged (V+C). Vaccination was performed using irradiated APEC applied *via* aerosol. For challenge, the homologous strain was administered intratracheally. Birds were sacrificed on 3, 7, 14 and 21 days post challenge (dpc) to examine lesions, organ to body weight ratios and bacterial colonization. Lung and spleen were sampled for investigating gene expression of cytokines mediating inflammation by RT-qPCR and changes in the phenotype of subsets of mononuclear cells by flow cytometry. After re-stimulation of immune cells by co-cultivation with the pathogen, APEC-specific IFN-γ producing cells were determined. Challenged only birds showed more severe pathological and histopathological lesions, a higher probability of bacterial re-isolation and higher organ to body weight ratios compared to vaccinated and challenged birds. In the lung, an upregulation of *IL-1β* and *IL-6* following vaccination and/or challenge at 3 dpc was observed, whereas in the spleen *IL-1β* was elevated. Changes were observed in macrophages and TCR-γδ^+^ cells within 7 dpc in spleen and lung of challenged birds. Furthermore, an increase of CD4^+^ cells in spleen and a rise of Bu-1^+^ cells in lung were present in vaccinated and challenged birds at 3 dpc. APEC re-stimulated lung and spleen mononuclear cells from only challenged pullets showed a significant increase of IFN-γ^+^CD8α^+^ and IFN-γ^+^TCR-γδ^+^ cells. Vaccinated and challenged chickens responded with a significant increase of IFN-γ^+^CD8α^+^ T cells in the lung and IFN-γ^+^TCR-γδ^+^ cells in the spleen. Re-stimulation of lung mononuclear cells from vaccinated birds resulted in a significant increase of both IFN-γ^+^CD8α^+^ and IFN-γ^+^TCR-γδ^+^ cells. In conclusion, vaccination with irradiated APEC caused enhanced pro-inflammatory response as well as the production of APEC-specific IFN-γ-producing γδ and CD8α T cells, which underlines the immunostimulatory effect of the vaccine in the lung. Hence, our study provides insights into the underlying immune mechanisms that account for the defense against APEC.

## 1 Introduction

Avian pathogenic *Escherichia coli* (APEC) is an opportunistic and primary pathogen of chickens, which causes colibacillosis. Colibacillosis is a complex of respiratory and systemic diseases that is considered a substantial welfare issue responsible for significant economic losses of poultry production worldwide due to high morbidity, mortality and carcass condemnation (1). The disease can affect all age groups of chickens (1, 2). In commercial layers, peritonitis, salpingitis, oophoritis, arthritis, osteomyelitis, pneumonia and airsacculitis have been described (1). The respiratory route is accepted as the main infection pathway in conventional layers (3).

For combating colibacillosis, effective strategies are highly warranted to prevent and treat the disease in poultry production. Vaccination is one of these strategies which is essential to limit the disease development and the need for antibiotics (4). An ideal vaccine against colibacillosis is expected to have effective impacts on preventing colonization and destruction of organs by the pathogen. Furthermore, it should reduce mortality and establish long term protective immunity in laying hens. Different vaccines, including live-attenuated, recombinant and subunit as well as autogenous vaccines have been developed to protect chickens against APEC infections (1). Among the tested vaccines, bacterial ghost vaccines, recombinant *iss*, recombinant antigen vaccine containing *E. coli* antigens, *Salmonella*-delivered vaccines containing APEC antigens, outer membrane vesicles, siderophore receptor and porin (SRP), genes deleted mutants (*aroA*, *crp* and *tonB/fur*) or gamma-irradiated *E. coli* have shown wide range of protections (1, 2, 5). Several routes such as oral, aerosol or intramuscular, were used for the delivery of *E. coli* vaccines (6). As reviewed before, it was stated that challenge models that mimic the natural course of infection such as aerosolization of APEC would provide a better assessment of the efficacy of APEC vaccines (6). Despite plenty of vaccine candidates demonstrating efficacy in reducing mortality, lesions, and bacterial load, as well as to stimulate antibody responses in chickens in experimental studies, only a few vaccines are currently commercially available (2, 7) which emphasizes a merit of presenting novel approaches to effectively control APEC infections in poultry.

Recently, interest in the application of ionizing radiation to inactivate pathogens for vaccine production increased significantly in veterinary medicine, including poultry (8). Among the ionizing-radiation vaccines, a gamma irradiated APEC vaccine demonstrated to provide protection against colibacillosis (5). Gamma radiation inactivates microorganism by ionization. Thereby, the DNA is targeted by the ionizing radiation and the removal of electrons from the atoms of the large molecules resulting in inactivation while the cellular membrane remains intact (8). As a result, the microbial cells stop to multiply due to damage of their nucleic acids but they are able to maintain their metabolic activities (8, 9). It was shown that killed but metabolically active (KBMA) *Listeria monocytogenes*, inactivated by ultraviolet irradiation, keeps the biologic properties of the parental organism, and thus will stimulate host immune response (10). Similar findings have been demonstrated for irradiated *E. coli* cells which exhibit characteristics of live, non-irradiated cells, such as intact membrane integrity and metabolic activity, but are unable to undergo replication (5, 11). However, a detailed picture of the correlation between the immune response and protection after vaccination with an irradiated APEC vaccine is largely unknown.

Generally, the current knowledge about the immune response against APEC is limited as there are only few studies which primarily focused on bacteriological and histopathological examinations of the affected organs such as lung and changes of antibody levels (12). The immunological basis of protection against APEC as a primary pathogen is described in the current literature which revealed that a pro-inflammatory response and cellular innate immune response play a role in this context (13). Previous transcriptome studies analysing lung, bone marrow, thymus, bursa, spleen or blood derived leukocytes showed an important function of the innate immune response to APEC (14–16). As it was reviewed before, in regard to cellular innate immunity, heterophils, macrophages, dendritic cells and thrombocytes are described to be important in the defense against APEC (13). Innate immune cells recognize distinct pathogen-associated molecular pattern (PAMP) on the bacterial surface, which in turn activates intracellular signaling pathways resulting in antimicrobial activities and the production of pro- and anti-inflammatory cytokines. In the context of APEC mounting and coordinating, an immune response depends on various cytokines, particularly *IL-1β* and *IL-6* which activate and regulate immune cells during host-pathogen interaction (17–23). Unlike young chickens in which immunity largely depends on activity of the innate immune system as well as on maternal antibodies, in adult birds the adaptive immune system is fully developed and functional T and B cell responses can be expected (24). Although, the development and role of immune cells regarding an APEC infection in chicken is highlighted (25) little has been published explaining the dynamics of the immune system of *E. coli* infected chickens using flow cytometry on samples of circulating cells such as monocytes/macrophages, CD4^+^ or CD8^+^ cells (17, 26, 27). Recently, investigations on cytokine production of T cells after *ex vivo* re-stimulation revealed significantly elevated levels of APEC-specific IFN-γ-producing CD8α, CD4, TCR-γδ cells in lung, spleen and blood mononuclear cells of naïve specific pathogen-free (SPF) chickens (28). However, the APEC-specific IFN-γ-producing T-cell response of primed mononuclear cells following vaccination and/or challenge with APEC was not addressed and remains unexplored.

In this study, clinical, pathological and bacteriological outcomes in context of vaccination and/or challenge with a homologous strain of APEC in commercial pullets were examined. Then, we investigated how live or irradiated APEC affects innate and adaptive immune cells in lung and spleen to obtain more insight in the contribution of the immune system against infection in context of vaccination. Cytokine gene expression was determined alongside differences in kinetics of immune cells subsets between unchallenged, vaccinated and/or challenged birds. The subsequent adaptive responses were determined including presence and activation of T-cell subsets and serum antibody levels. Hence, the present study aimed to provide information on local and systemic immune responses that are evoked by irradiated and live APEC in chickens.

## 2 Materials and methods

### 2.1 Inoculum and vaccine preparation

In the present work the well-characterized field isolate APEC PA14/17480/5-ovary (serotype O1:K1) was used for vaccine and challenge preparations. The strain was isolated from the ovary of an infected chicken suffering from egg peritonitis, perihepatitis and salpingitis (29) and characterized *in vitro* (30). For the preparation of the challenge culture, a bacterial suspension in 10 ml of Lenox L Broth Base (LB, Invitrogen, Vienna, Austria) was made by cultivation at 37°C with agitation for 18 h under aerobic conditions. After that, 50 µl of bacterial suspension were transferred to 25 ml of LB broth and incubated for 6 h (37°C, 150×rpm). Following incubation, the bacterial culture was washed two times and resuspended in phosphate buffered saline (PBS) (Gibco™, Thermo Fisher Scientific, Waltham, USA). The culture was then mixed with 25 ml of PBS, resulting in a final volume of 50 ml, which was used as inoculum for challenge. This protocol for the growth and preparation of bacterial cultures yielded in a concentration of 8 log_10_ CFU/ml of bacteria determined by counting colony-forming units (CFU) of serially diluted suspensions in duplicate. For preparation of the gamma-irradiated APEC vaccine, separate cultures of bacteria were prepared as described above with a slight modification. At the final step, after washing and resuspension of the bacterial pellet in PBS, 25 ml of Trehalose solution (1M) (Carl Roth GmbH, Karlsruhe, Germany) was added. The irradiation was done at the International Atomic Energy Agency Laboratories in Seibersdorf, Austria. Samples incubated on ice were irradiated using a Model 812 Co-60 irradiator at a dose rate of 66.532 Gy/min (Foss Therapy Services, Inc., California, USA). The irradiator was regularly calibrated using an ionization chamber that also mapped the delivered dose in the location where the samples are irradiated. A gamma ray dose 1.2 kGy was applied based on a prior dose titration experiment that was able to inactivate the bacteria without losing their metabolic activity. Thus, the time spent for inactivation was 18 minutes. To confirm that the irradiated APEC lost their replication capability, 1 ml of the cell solution was cultivated overnight with 5 ml of LB broth and subsequently plated on MacConkey agar plates (Neogen, Heywood, UK). The plates were incubated at 37°C and the growth of colonies was evaluated. In this way, the loss of growth ability of irradiated bacterial cells was confirmed. The irradiated bacteria were kept at 4°C until further use as vaccine within 18 h.

### 2.2 Birds and experimental design

**Figure 1:**
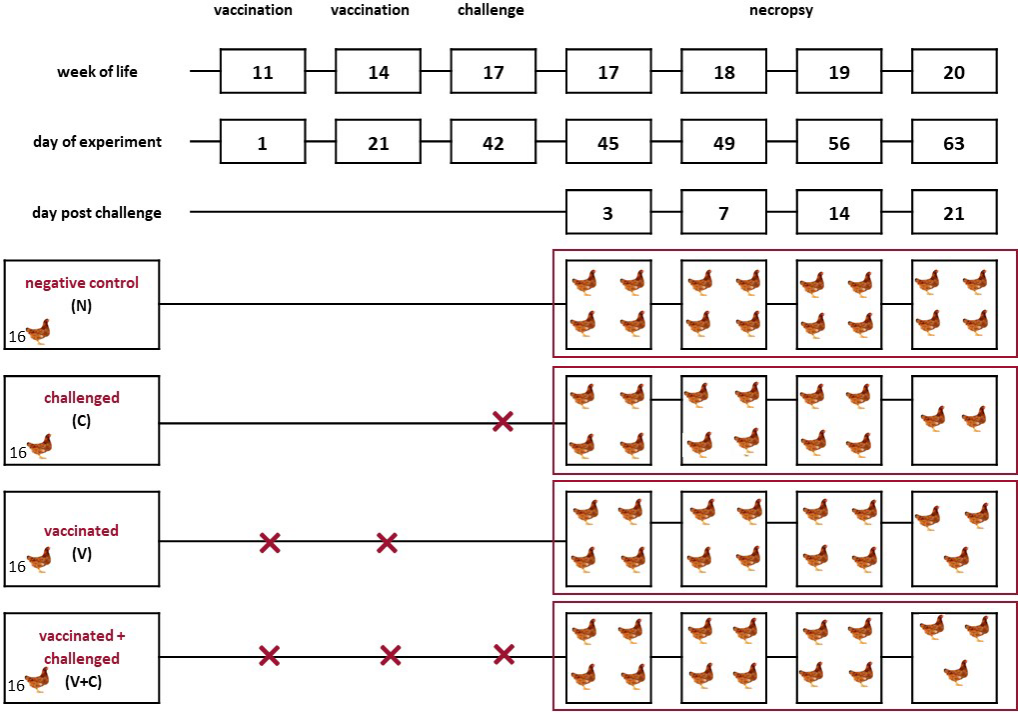
Schematic representation of the experimental setup. Pullets (n = 16 per group) from the negative control (N), challenged (C), vaccinated (V), vaccinated and challenged (V+C) groups were kept in separate isolators from the first day of the experiment. Two birds of group C, one bird of group V+C and one bird in group V died or had to be euthanized before predetermined sampling time points. The vaccination was performed using an irradiated APEC strain applied *via* aerosol. For challenge, the virulent, homologous strain was administered intratracheally.

Commercial layer pullets (Lohmann Brown, Lohmann Breeders, Cuxhaven, Germany) without any history of colibacillosis were purchased from a conventional Austrian farm (Schropper GmbH, Gloggnitz, Austria). All birds were vaccinated against Newcastle disease, infectious bronchitis, infectious bursal disease, Marek’s disease, *Salmonella* Enteritidis and coccidiosis. At the age of 10 weeks, sixty-four pullets were transferred from a commercial rearing farm to the experimental facilities of the Clinic for Poultry and Fish Medicine, University of Veterinary Medicine Vienna, Austria. Birds were divided randomly into four groups with 16 birds each. A schematic representation of the experimental setup is shown in the **Figure 1**. Each group of birds was placed in a separate isolator (Montair Andersen bv, Sevenum, The Netherlands) under negative pressure. The birds were kept in the isolators for 7 days without any intervention in order to acclimatize them to their new environment. A subcutaneous tag was applied to every bird for identification (Swiftag^®^, Avery Dennison, Glendale, USA). After that, birds from the “vaccinated” and “vaccinated and challenged” groups (group V and group V+C, respectively) were vaccinated *via* aerosol route two times at an interval of 3 weeks. Likewise, birds in the “challenged only” and “negative control” groups (group C and group N, respectively) received the same volume of PBS. For aerosolization, the airflow in the isolators was switched off and 5 ml vaccine suspension or PBS per bird were nebulized inside. The set up was similar as previously described (31) but different equipment for aerosolization of a larger volume were used. Thereby, a nozzle (Frans Veugen Bedrijfshygiëne B.V., Nederweert, The Netherlands) was applied that generated a breathable fraction of 5 µm droplets. The air compressor (Airpress, Erpolzheim, Germany) was adjusted to deliver a pressure of 2.5 bar. The specific pressure was chosen based on the calibration report provided by the supplier. Nebulisation time was set to 10 minutes. Following nebulization, 30 minutes of ventilation was upheld to ensure delivery of vaccine particles. Three weeks after the second vaccination, birds from groups C and V+C were challenged. In total, 1 ml (8 log 10 CFU/ml) cultivated bacteria per bird was administered *via* the intratracheal route. The concentration of the bacteria was selected according to previous studies (31, 32). For birds in groups N and V, PBS was administered in the same way. During the whole trial, light duration was set to 8 hours per day and feed and water were provided *ad libitum*.

### 2.3 Clinical examination, necropsy and sampling

All birds were examined daily for clinical signs related to colibacillosis including weakness, dropped wings, depression, ruffled feathers, reluctance to move, apathy, ability to move or stand, closed eyes and intensified breathing. From the first week of the experiment onwards, blood was collected weekly to obtain serum for an indirect ELISA detecting APEC specific antibodies (immunoglobulins (Ig) Y) as previously reported by Paudel et al. (5).

Before the experiment, 4 predetermined birds from each group were selected to be sacrificed according to their individual tag number in ascending order at 3, 7, 14 and 21 days post challenge (dpc) for necropsy and sampling. Birds that died after infection were also submitted to pathological examination. Euthanasia was performed by intravenously administered thiopental (Medicamentum pharma GmbH, Allerheiligen im Mürztal, Austria) and subsequent bleeding to death. During the ensuing necropsy, lesions of the affected organs were scored. For that, an already established scoring system of pathological changes in organs was applied with some modifications (**Supplementary Table 1**) (33). During necropsy, total body weight (BW) and weights of lung, spleen and liver from all birds were recorded. The weight of the respective organs in proportion to the body weight of each bird was calculated as: (weight of organ/body weight) × 100%. For bacterial detection in lung, spleen, liver, heart and air sac, samples were collected from all birds during necropsy and plated on MacConkey agar (Neogen, Heywood, UK). For histological preparations, tissue samples from lung, spleen, liver and heart were preserved in 10% neutral buffered formalin. For RNA extraction, samples from lung and spleen were taken, placed in RNAlater^®^ (Qiagen, Hilden, Germany) and stored at −80 °C until further use. For isolation of mononuclear cells, lungs and spleens were placed in ice-cold PBS + 2% fetal calf serum (FCS) (both Gibco™, Thermo Fisher Scientific) filled beakers until they were processed immediately after necropsy.

### 2.4 Histological evaluations

Formalin fixed samples from lung, spleen, liver and heart were washed and dehydrated before embedded in paraffin. The samples were taken from all birds in groups C and V+C and also from two birds per time point with lowest identification numbers from groups N and V. The samples were sectioned at 5 μm thickness with a microtome (Microm HM 360, Microm Laborgeräte GmbH, Walldorf, Germany) and mounted on glass slides for being stained with hematoxylin and eosin (HE). In order to investigate the colonization of tissues by *E. coli*, paraffin embedded samples were processed for immunohistochemistry (IHC). The IHC protocol for the detection of *E. coli* was followed as described previously by Abdelhamid et al. (34). Briefly, tissue sections mounted on charged glass slides were dewaxed, rehydrated and incubated overnight with a primary monoclonal antibody (anti-*E. coli* LPS antibody (2D7/1), Abcam, Cambridge, UK). Following incubation, the slides were washed with PBS and biotinylated anti-mouse IgG antibody (Vector Laboratories, Burlingame, USA) was added. Then, the vectastain ABC Kit and DAB substrate kit (Vector Laboratories) were used for visualization of the bound antibodies. Finally, the sections were counter-stained with Mayer’s haematoxylin (Merck KGaA, Darmstadt, Germany) and observed under microscope. Examination was performed using the Olympus BX53 microscope equipped with an Olympus DP72 camera (Olympus Corporation, Tokyo, Japan) for documentation.

### 2.5 Gene expression by RT-qPCR

For total RNA extraction, 30 mg from the organ samples stored in RNAlater^®^ (Qiagen) were individually homogenized using Tissue Lyser (Qiagen, Hilden, Germany) (2x at 30 Hz for 2 min). RNA extraction was performed by RNeasy^®^ mini kit (Qiagen) according to manufacturer’s instructions. The purity and quantity of extracted RNA was analysed by NanoDrop 2000 (ThermoFisher scientific) and Qubit™ fluorometers (Invitrogen). The RNA quality of every sample was evaluated by 4200 TapeStation system (Agilent Technologies, Waldbronn, Germany) to determine the RNA integrity number (RIN). RNA samples with RIN values above 8 were considered for reverse transcriptase quantitative polymerase chain reaction (RT-qPCR) analysis, otherwise extractions were repeated to reach the mentioned RIN value. Previously published primers and probes were used for targeting the reference genes TFRC (transferrin receptor protein), TBP (TATA box binding protein) (35) together with the cytokines *IL-1β*, *IL-6*, *IL-10* and *IFN-γ* (**Supplementary Table 2**) (36). The expression levels of cytokines mRNA were quantified by RT-qPCR using Brilliant III Ultra-Fast qRT-PCR master mix kit (Agilent Technologies). To amplify the primary transcripts and quantify their specific products, AriaMx real-time PCR system (Agilent Technologies) and Agilent AriaMx1.7 software (Agilent Technologies) were used. All samples were run in duplicate. The thermal cycle profile was as followed: 1 cycle of reverse transcription at 50°C for 10 min followed by 95°C for 3 min to hot start, 40 cycles of amplification at 95°C for 5 s and 60°C for 30 s. Different types of controls, including non-reverse transcriptase and non-template control were run to ensure that samples were free from genomic DNA contamination and overall PCR contamination, respectively. The RT-qPCR investigation followed the MIQE guidelines (37). To account for the variation in sampling and RNA preparation, the Cq values for all genes were normalized using Cq values of previously reported reference genes *TFRC* and *TBP* (35). The normalised mean Cq value of each cytokine was then used to calculate the fold change from each group by applying the formula 2^(−ΔΔCq)^ (38). The average of Cq values of examined lung and spleen of each bird is presented supplementary (**Supplementary Table 3**). For graphical representation of data, vaccinated and/or challenged groups were adjusted with the control group and given as fold change value.

### 2.6 Flow cytometry

#### 2.6.1 Isolation of mononuclear cells

Mononuclear cells were isolated according to a previously described protocol for the lung and spleen (28). Briefly, single cells from spleens were obtained by mechanical dissection using two sterile blunt-end forceps. For the lung, the tissue was cut into small pieces and enzymatically digested for 30 min in Dulbecco’s Modified Eagle Medium (DMEM) +10% FCS (both Gibco™, Thermo Fisher Scientific) with 0.5 mg/ml collagenase type IV (Sigma-Aldrich St. Louis, USA) at 41°C with stirring. The cells were then separated from the remaining tissue through a 40 µm nylon cell strainer (BD Falcon, BD Bioscience, San Jose, USA). The isolated cells were centrifuged at room temperature at 350×g for 10 min. Following resuspension of the cell pellet in cold PBS+2% FCS, the cell suspension was layered on a double volume of Histopaque^®^-1077 (Sigma-Aldrich). The cells from the interphase layer were collected after centrifugation at 850×g for 20 min at room temperature. After two washing steps (350×g, 10 min, 4°C) with PBS+2% FCS the cells were re-suspended in 1 ml PBS+10% FCS. A final concentration of 2 × 10^7^ live cells/ml of PBS+2% FCS was adjusted for the latter described experiments.

#### 2.6.2 Phenotypic characterization of directly isolated mononuclear cells

Multicolor staining was applied with isolated mononuclear cells from lung and spleen samples of the birds. Panel matrices were designed firstly to target live cells and then populations of immune cells positive for CD45, CD4, CD8α, TCR-γδ and B cells as well as monocytes/macrophages of chicken. Antibodies and antibody combinations used to determine immune cell populations are presented in **Supplementary Table 4**. The concentration of each antibody was determined through titration and the corresponding isotype controls were included. To stain the mononuclear cells isolated from lung and spleen, 20 µl of the adjusted cell suspension was transferred into wells of 96-well microtiter plates (Sarstedt, Nümbrecht, Germany) along with the respective primary antibodies. The cells were then incubated for 20 min at 4°C. Afterwards, cell pellets were collected using centrifugation at 4°C, 350g for 4 min and washing was applied for two times with cold PBS+2% FCS. In a secondary staining step, Streptavidin eFluor™ 510 (Thermo Fisher Scientific) and BV421 (Jackson Immuno Research, West Grove, USA) were used combined with BD Horizon™ Fixable Viability Stain 780 (BD Biosciences) (20 min, 4°C). Following another incubation step for 20 min at 4°C further washing was performed. Finally, the pellets were re-suspended in 200 µl cold PBS+2% FCS and kept at 4°C until flow cytometry (FCM) analysis.

#### 2.6.3 Re-stimulation of cells and intracellular cytokine staining

An already established *ex vivo* model was applied to perform a re-stimulation experiment with mononuclear cells from lung and spleen samples (28). Isolated cells were separately seeded in 96-well round-bottom microtiter plates (Sarstedt, Nümbrecht, Germany). Per sample, 5 × 10^5^ cells/well in 200 µL RPMI 1640 Medium GlutaMAX™ Supplement and 10% FCS (both Gibco™, Thermo Fisher Scientific) were used and kept unstimulated or treated with irradiated APEC as well as PMA/ionomycin. For stimulation with irradiated APEC the mononuclear cells to APEC ratio 1:100 was used. As positive control, one set of wells was treated with 20 µl of PMA (50 ng/mL, Sigma-Aldrich) and ionomycin (500 ng/mL, Sigma-Aldrich) while culture medium was added to the negative control. The samples were incubated in a humidified incubator at 41°C and 5% CO_2_ for 6 h. Brefeldin A (BD GolgiPlug™, BD Biosciences) was used at a concentration of 1 µg/mL to inhibit cytokine secretion.

To perform surface staining, cells were incubated with mouse anti-chicken CD45, CD8α, CD4, TCR-γδ cells and monocyte/macrophage monoclonal antibodies for 20 min at 4°C (**Supplementary Table 4**). In a subsequent staining step, Streptavidin eFluor™ 510 (Thermo Fisher Scientific) and BV421 (Jackson Immuno Research) were applied along with BD Horizon™ Fixable Viability Stain 780 (BD Biosciences) for 20 min at 4°C. Afterwards, the cells were washed twice with PBS+2% FCS (both Gibco™, Thermo Fisher Scientific). Subsequently, the cells were fixated, permeabilized and stained for cytokine IFN-γ (clone: 12F7, isotype: IgG2a) intracellularly. After washing with the Perm/Wash™ Buffer (BD Biosciences), an incubation with goat anti-mouse IgG2a-RPE (Southern Biotech, Birmingham, USA) was performed. Following two washing steps, the cells were re-suspended in 200 µl Perm/Wash™ Buffer (BD Biosciences) solution for subsequent flow cytometry analysis.

#### 2.6.4 Cell analysis by flow cytometry

FCM of stained cells was performed on a FACSCanto II (BD Biosciences, San Jose, USA) flow cytometer equipped with three lasers (405, 488 and 633 nm). Analysis of FCM raw data was performed by FACSDiva software version 9 (BD Biosciences). For phenotypic characterization of mononuclear cells, at least 50,000 live leukocytes (CD45^+^ cells) per sample were recorded. Different gating strategies were applied (**Supplementary Figure 1**). A uniform gating hierarchy was used throughout all sampling days and for both organs. For IFN-γ producing cells, at least 200,000 live leukocytes (CD45^+^ cells) per sample were recorded. For all analyzed cell subsets, IFN-γ gates were set individually per bird within the unstimulated medium sample and applied to the stimulated samples (**Supplementary Figure 2**).

### 2.7 Statistical analysis

Effects on the log10-transformed organ to BW ratio and the arcsine-square-root-transformed frequencies of IFN-γ-producing cells were evaluated *via* one-way ANOVA in R (function *lm*) (39). The pattern of bacteria presence or absence in organs was treated as a binary response (negative = 0, positive = 1) and analyzed with a binary logistic regression model in R (function *glm*). In these models, the group was treated as a fixed categorical effect. Overall significance of the group was evaluated *via* a likelihood ratio test using the function *anova* in R, comparing the full model to a reduced model without the group as a fixed categorical effect. Pairwise comparisons between groups were conducted *via* the estimated marginal means (emmeans, package *emmeans*, function *emmeans*) (40).

Effects on mRNA gene expression and log10-transformed cells frequencies were evaluated *via* linear mixed models in R (package *lme4*, function *lmer*) (41). In these models, the interaction between group and time point was treated as a fixed categorical effect while the bird identification number (ID) was treated as a random effect to account for the covariance structure in the data, as each bird was examined twice [~ group * timepoint + (1 | birdID)]. Overall significance of the interaction was evaluated *via* a likelihood ratio test using the function *anova* in R, comparing the full model to a reduced model without the interaction. Multicolinearity between group and time point was assessed *via* the variance inflation factor (package *car*, function *vif*) (42). Pairwise comparisons against group N only for each time point were conducted *via* the estimated marginal means (emmeans, package *emmeans*, function *emmeans*) (40).

For all linear models and linear mixed models, the assumptions about the residuals and random effects (if present) were evaluated and met. For all the aforementioned analyses, significance was declared at an alpha cut off of 5% after multiple testing correction (using the Bonferroni-Holm method), as each analysis included multiple pairwise comparisons between groups. During statistical analysis, for organ to BW ratio and probability of bacterial detection analysis, both dead and sacrificed birds from all groups were included. For RT-qPCR and FCM analysis, only data of killed birds that were predetermined for sampling were included.

## 3 Results

### 3.1 Clinical signs and post-mortem

The onset of clinical signs started 1 dpc (**Table 1**). At this time point, two birds of group C showed mild depression reflected by dropped wings and half-closed eyes. The same birds showed acute progression of clinical signs at 3 dpc (severe depression, loss of appetite, closed eyes, reluctance to move) and died. In group V+C clinical signs started 1 dpc in two birds of which one chicken recovered fully within four days whereas the other bird had to be euthanized at 10 dpc. No clinical signs were recorded in the remaining chickens with the exception of one bird in group V which was euthanized because of cannibalism. Infection with APEC did not affect the weight of the pullets, as growth curves of all groups were very similar (data not shown).

**Table 1:** Clinical signs, pathological lesion scores, histopathological examination with detection of *E. coli* by immunohistochemistry and bacterial re-isolation from challenged birds (groups C and V+C).

Group	Bird identification	Day post challenge (dpc)	Occurrence of clinical signs (dpc)	Lung				Spleen			
				Lesion score	Bacterial isolation	Infiltration of inflammatory cells	IHC^a^	Lesion score	Bacterial isolation	Infiltration of inflammatory cells	IHC
**Negative control**	1	3	–	0	+	–	–	0	–	–	–
	2	3	–	0	–	–	–	0	–	–	–
	3	3	–	0	–	n.d^b^	n.d	0	–	n.d	n.d
	4	3	–	0	–	n.d	n.d	0	–	n.d	n.d
	5	7	–	0	–	–	–	0	–	–	–
	6	7	–	0	–	–	–	0	–	–	–
	7	7	–	0	+	n.d	n.d	0	+	n.d	n.d
	8	7	–	0	+	n.d	n.d	0	–	n.d	n.d
	9	14	–	0	+	–	–	0	–	–	–
	10	14	–	0	+	–	–	0	+	–	–
	11	14	–	0	–	n.d	n.d	0	–	n.d	n.d
	12	14	–	0	–	n.d	n.d	0	–	n.d	n.d
	13	21	–	0	–	–	–	0	–	–	–
	14	21	–	0	–	–	–	0	–	–	–
	15	21	–	0	–	n.d	n.d	0	–	n.d	n.d
	16	21	–	0	–	n.d	n.d	0	–	n.d	n.d
**Challenged**	17	3	–	1	+	+	–	0	+	–	–
	18	3	–	2	+	+	+	0	–	+	+
	19 ^c^	3	1-3	2	+	+	+	1	+	+	+
	20	3	–	1	+	+	–	1	+	–	–
	21	3	–	2	+	+	+	1	–	–	–
	22	7	–	0	+	+	–	0	–	–	–
	23 ^c^	3	1-3	2	+	+	+	1	+	+	+
	24	7	–	0	+	–	–	0	–	–	–
	25	7	–	2	+	+	+	0	–	–	–
	26	7	–	0	+	–	–	0	–	–	–
	27	14	–	0	–	–	–	0	–	–	–
	28	14	–	0	+	–	–	0	–	–	–
	29	14	–	1	+	+	–	0	–	–	–
	30	14	–	0	–	–	–	0	–	–	–
	31	21	–	1	–	–	–	0	–	–	–
	32	21	–	0	–	+	–	0	–	+	+
**Vaccinated**	33	3	–	0	+	–	–	0	–	–	–
	34	3	–	0	–	–	–	0	–	–	–
	35	3	–	0	–	n.d	n.d	0	–	n.d	n.d
	36	3	–	0	+	n.d	n.d	0	–	n.d	n.d
	37	7	–	0	–	–	–	0	–	–	–
	38	7	–	0	–	–	–	0	–	–	–
	39	7	–	0	+	n.d	n.d	0	–	n.d	n.d
	40	7	–	0	+	n.d	n.d	0	–	n.d	n.d
	41	14	–	0	–	–	–	0	–	–	–
	42	14	–	0	–	–	–	0	–	–	–
	43	14	–	0	–	n.d	n.d	0	–	n.d	n.d
	44 ^d^	10	–	0	–	n.d	n.d	0	–	n.d	n.d
	45	14	–	0	+	n.d	n.d	0	–	n.d	n.d
	46	21	–	0	–	–	–	0	–	–	–
	47	21	–	0	–	–	–	0	–	–	–
	48	21	–	0	–	n.d	n.d	0	–	n.d	n.d
**Vaccinated and challenged**	49	3	–	1	–	–	–	0	–	–	–
	50	3	–	2	+	+	+	0	–	–	–
	51	3	–	0	+	+	+	0	+	+	+
	52	3	–	2	+	–	–	0	+	–	–
	53	7	1-4	2	+	+	–	0	–	–	–
	54	7	–	0	–	–	–	0	–	–	–
	55	7	–	0	+	–	–	0	–	–	–
	56	7	–	2	+	+	+	0	–	–	–
	57	14	–	0	–	–	–	0	–	–	–
	58	14	–	0	–	–	–	0	–	–	–
	59	14	–	2	+	+	+	0	+	–	–
	60	14	–	0	+	+	–	0	–	–	–
	61	21	–	0	–	–	–	0	–	–	–
	62	21	–	0	–	–	–	0	–	–	–
	63	21	–	0	–	–	–	0	–	–	–
	64 ^c^	10	1-10	2	+	+	+	0	+	–	–

a detection of bacteria was performed by immunohistochemistry

b not done

c bird that died or had to be euthanized due to severe clinical signs

d bird that had to be euthanized due to cannibalism

Only vaccinated and negative control birds (groups V and N) did not show clinical signs, lesions scores or histological changes related to colibacillosis.

**Figure 2:**
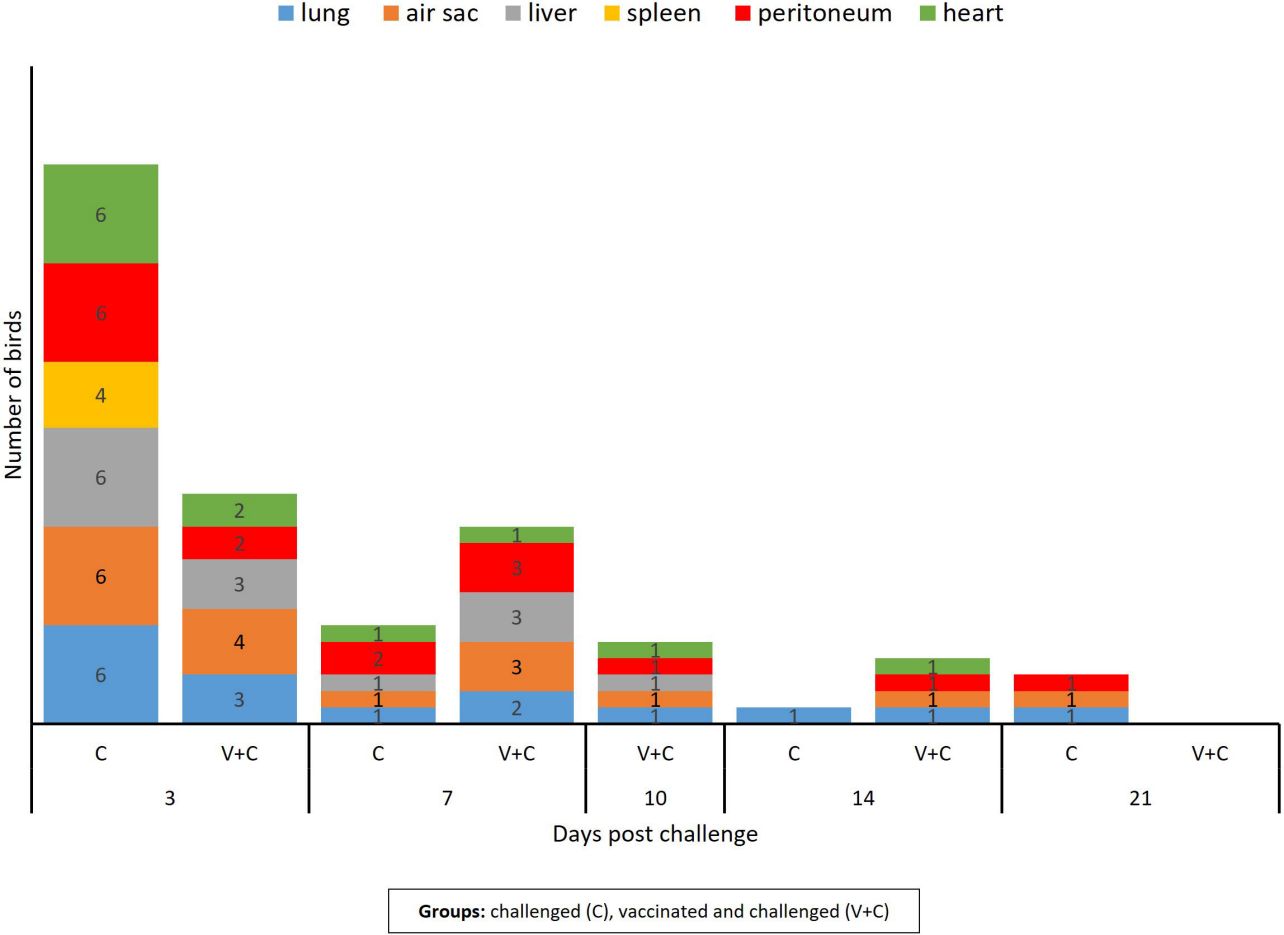
Number of birds with lesion in lung, spleen, liver, air sac, heart, and peritoneum following vaccination and/or challenge of birds. The column bar indicates the number of birds showing lesions in the respective organ at different time points. Chickens of the remaining groups (only vaccinated birds and negative control birds) did not show lesions related to APEC inoculation.

At necropsy, challenged birds (groups C and V+C) showed lesions such as airsacculitis, perihepatitis and pericarditis, consolidated lungs overlaying fibrinopurulent material and peritonitis. The cumulative number of organs with detectable lesions in heart, peritoneum, spleen, liver, air sac or lung was higher in group C (n=44) than in group V+C (n=35) (**Figure 2**). The mean of total lesion scores was 0.318 and 0.282 for groups C and V+C, respectively. Individual lesion scores of every examined lung and spleen of the challenged birds are listed in **Table 1** and changes in liver, air sac, heart and peritoneum are provided as supplement (**Supplementary Table 5**). None of the birds in groups N or V showed lesions at any sampling day.

**Figure 3:**
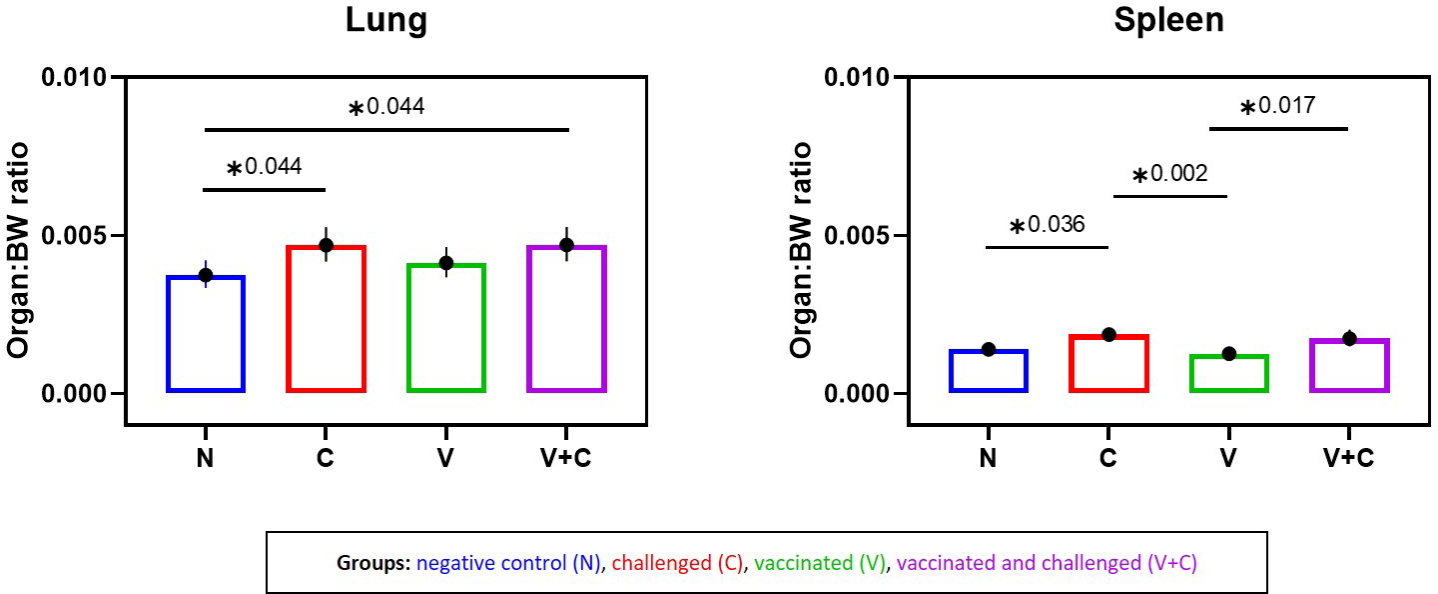
Organ to body weight (BW) ratios following vaccination and/or challenge of birds. Comparison of lung-BW ratio and spleen-BW ratio for each experimental group (negative control group (N), challenged group (C), vaccinated group (V), vaccinated and challenged group (V+C)) after challenge. Statistical differences were evaluated using one-way ANOVA in R. Significance was declared at an alpha cut off of 5%. Pairwise comparisons between groups were corrected for multiple testing *via* the Bonferroni-Holm method. Asterisks indicate a significant difference (*P* ≤ 0.05).

Average weights of lung and spleen to BW were affected (**Figure 3**). A significant increase in the lung/BW ratio was evident in groups V+C and C compared to group N (*P*=0.044). Furthermore, comparison between groups V+C and C, showed higher lung/BW ratio in group V+C (not significant). For the spleen, differences were observed in both challenged groups (V+C and C), with organ/BW ratios being significantly higher than in group V (*P*=0.017 and 0.002 respectively). In addition, spleen values from birds of group C were significantly higher than those from control birds (group N) (*P*=0.036). Comparing groups vaccinated and challenged (V+C) with challenged only (C), showed that the spleen/BW ratio was higher in group C than V+C however not reaching significance. For liver/BW-ratios, no significant differences were observed between groups (data not shown).

### 3.2 Bacteriology and serology

**Figure 4:**
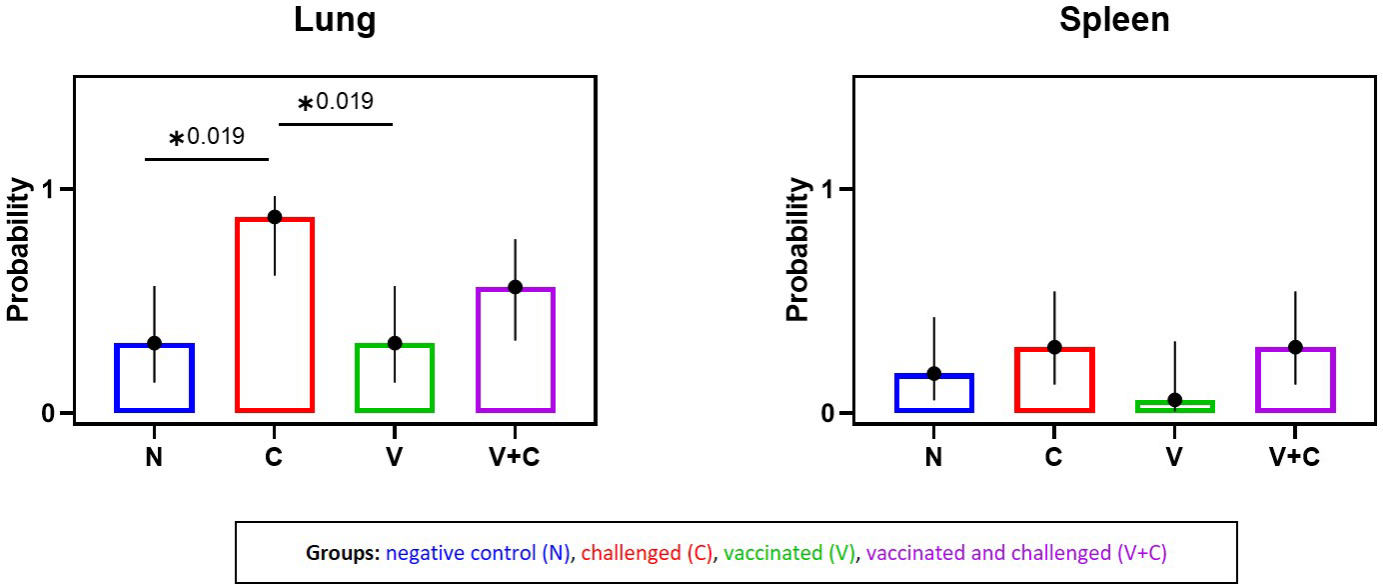
Predicted probability of bacterial isolation in lung and spleen following vaccination and/or challenge of birds. Comparison of probability of bacterial isolation between experimental groups (negative control group (N), challenged group (C), vaccinated group (V), vaccinated and challenged group (V+C)) in lung and spleen. Statistical differences were evaluated analyzed using a binary logistic regression model in R. Significance was declared at an alpha cut off of 5%. Pairwise comparisons between groups were corrected for multiple testing *via* the Bonferroni-Holm method. Asterisks indicate a significant difference (*P* ≤ 0.05).

Bacterial re-isolation was successful from all investigated organs revealing the systemic bacterial spread of APEC in challenged birds (**Table 1**, **Supplementary Table 5**). Five birds each in groups V and N, and 2 birds in group N were sampled positive in the lung and spleen, respectively. The likelihood of bacterial re-isolation was significantly different exclusively in the lung between the groups: Challenged birds showed a higher probability compared to pullets from groups V and N (**Figure 4**) (*P*=0.019). Comparing groups vaccinated and challenged (V+C) with challenged only (C), the likelihood of bacterial re-isolation was higher in lung, liver and air sac of group challenged only, however, not reaching significance. Data of liver, air sac and heart with no statistical significance are provided in **Supplementary Figure 3**.

Results from the testing for circulating antibodies against APEC in sera from birds did not differ between the groups at any sampling time point before and after challenge (mean optical density values ranged from 2.6 to 3.4). No significant variation in the concentration of IgY antibodies was seen in any group throughout the whole experiment (**Supplementary Figure 4**).

### 3.3 Histopathological examination and immunohistochemistry

**Figure 5:**
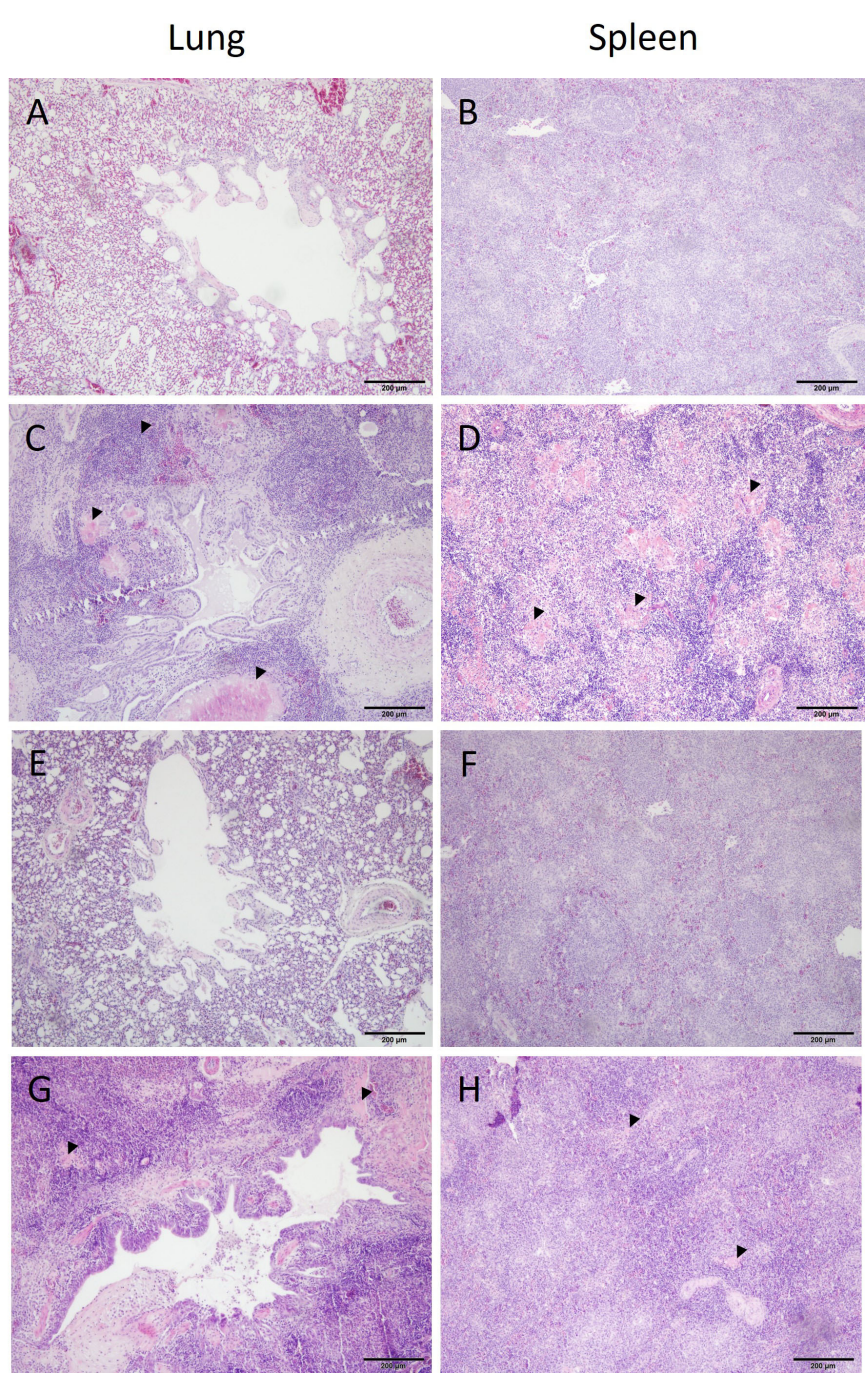
Histopathology of lung and spleen from different groups. Histopathological observations in tissues from a negative control (**(A, B)** bird identification: 1), challenged only (**(C, D)** bird identification: 19), vaccinated (**(E, F)** bird identification: 47) and vaccinated and challenged (**(G, H)** bird identification: 51) of representative birds are shown. Organ samples in **(C, D)** show multifocal areas of necrosis and severe infiltrations of leukocytes. Tissue sections in C and H indicate mainly an increased presence of inflammatory cells. Organ samples of the negative control and vaccinated birds do not show histopathological lesions.

Microscopic lesions were only seen in birds challenged with APEC (groups V+C and C) (**Table 1**). Inflammation was a typical feature demonstrated in organs of challenged pullets together with congestion and oedematous areas including fibrinous deposits. Samples from birds of group C presented infiltrations of inflammatory cells (heterophils and mononuclear cells) in the lung, spleen, liver, air sac and heart. Similar histological lesions were observed in the same organs of V+C birds, although substantially reduced compared to non-vaccinated but challenged birds (group C). Histopathological changes in lung and spleen from representative birds of all experimental groups are comparatively shown in **Figure 5**. No histopathological lesions were observed in organs of birds from groups N and V.

**Figure 6:**
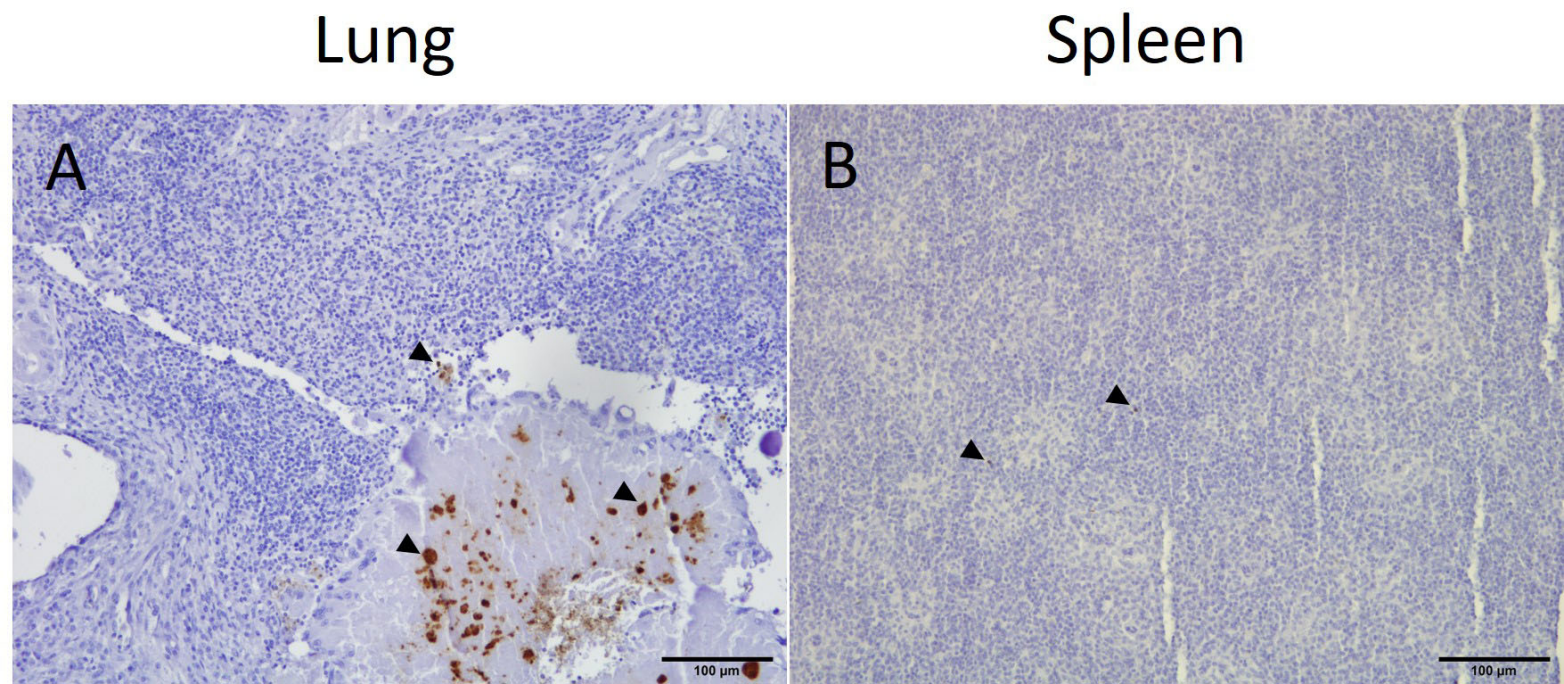
Immunohistochemical detection of *E*. *coli* in representative positive tissue samples. Presence of *E*. *coli* (arrow heads) in the lung **(A)** and spleen **(B)** of an APEC-challenged bird (bird identification: 19).

In samples from the lung and spleen, *E. coli* was only detected by IHC in birds that were inoculated with APEC (groups V+C and C) (**Table 1**). In the lung, *E. coli* was present in the capillary region and the larger lumina of the parabronchi of 5 and 4 out of 16 birds in groups C and V+C, respectively. Small colonies of bacteria were identified in necrotic areas. The presence of APEC was generally less common in spleen sections, however, bacteria were found in the parenchyma of 4 birds in group C and 1 bird in group V+C out of 16 birds in both groups. The localization and distribution of *E. coli* in the lung and spleen of a representative bird from group C is shown in **Figure 6**.

### 3.4 Gene expression of cytokines

**Figure 7:**
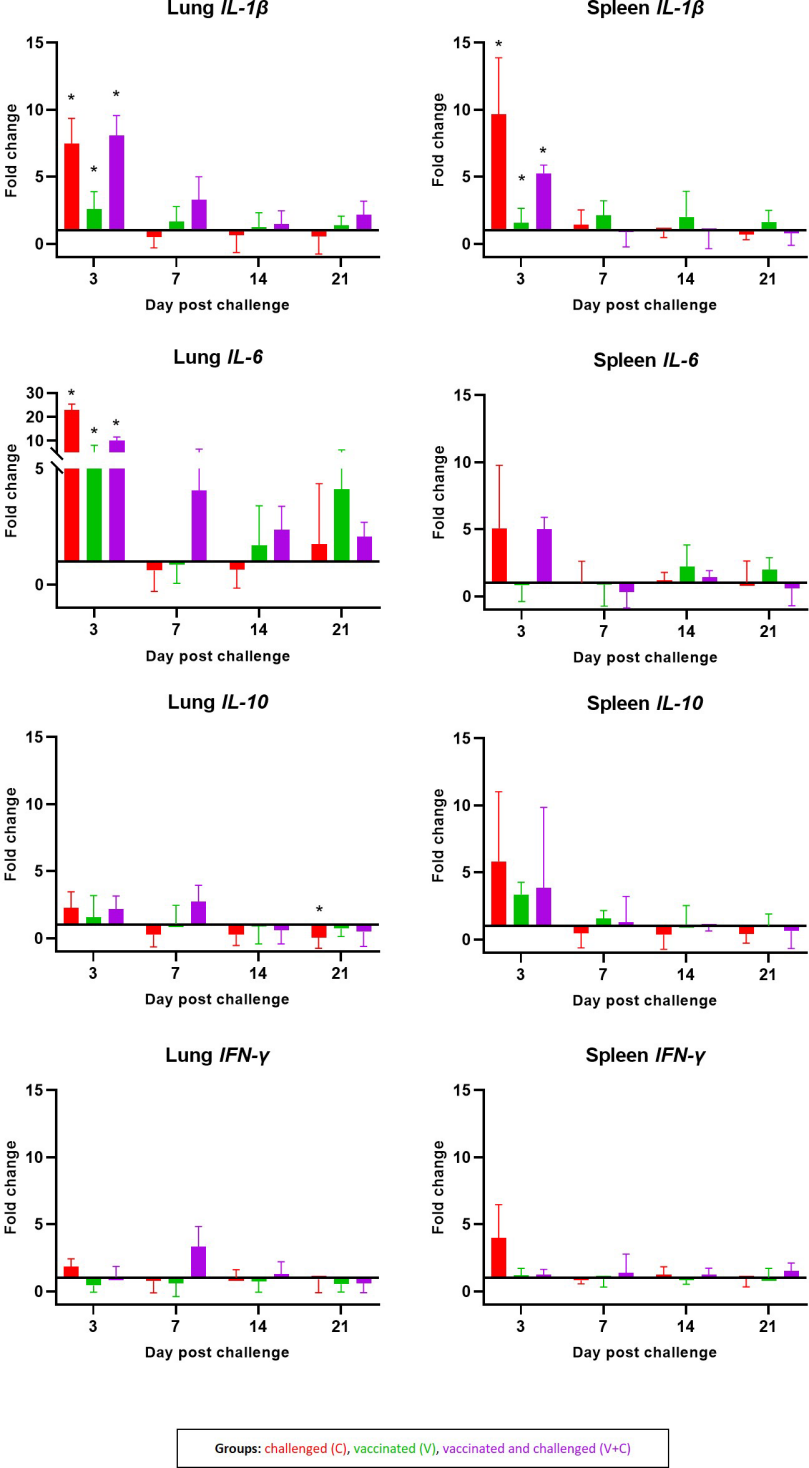
Cytokines mRNA expression levels measured by RT-qPCR. Expression pattern of *IL-1β*, *IL-6*, *IL-10* and *IFN-γ* in lung and spleen are shown as fold changes of mRNA expression levels at 3, 7, 14 and 21 dpc in groups C (red, n = 14) V (green, n = 15) V+C (purple n = 15). Statistical differences were calculated in comparison to the negative control group (n = 16). Error bars represent the standard error of ΔCq values. Statistical differences were evaluated using linear mixed model in R. Significance was declared at an alpha cut off of 5%. Pairwise comparisons between groups were corrected for multiple testing *via* the Bonferroni-Holm method. Significant changes are indicated as * (*P* ≤ 0.05).

Most frequent significant changes in mRNA gene expression for *IL-1β*, *IL-6*, *IL-10* and *IFN-γ* were observed at 3 dpc in the lung and spleen (**Figure 7**). At this time point following vaccination and/or challenge (groups C, V and V+C), mRNA levels for *IL-1β* were significantly upregulated in lung (*P*=0.0010, 0.0018 and 0.000001, respectively) and in spleen (*P*=0.0010, 0.0035 and 0.000002, respectively) in comparison to the control group. Furthermore, a significant upregulation in gene expression of *IL-6* in the lung was observed in birds of the same groups (*P*=0.0031, 0.0284 and 0.0060, respectively). At 21 dpc, mRNA levels for *IL-10* in lung samples were significantly lower in the group C in comparison to group N. No significant differences between the groups were found at any time point for *IFN-γ* neither in spleen nor lung samples.

### 3.5 Phenotypic characterization of mononuclear cells

**Figure 8:**
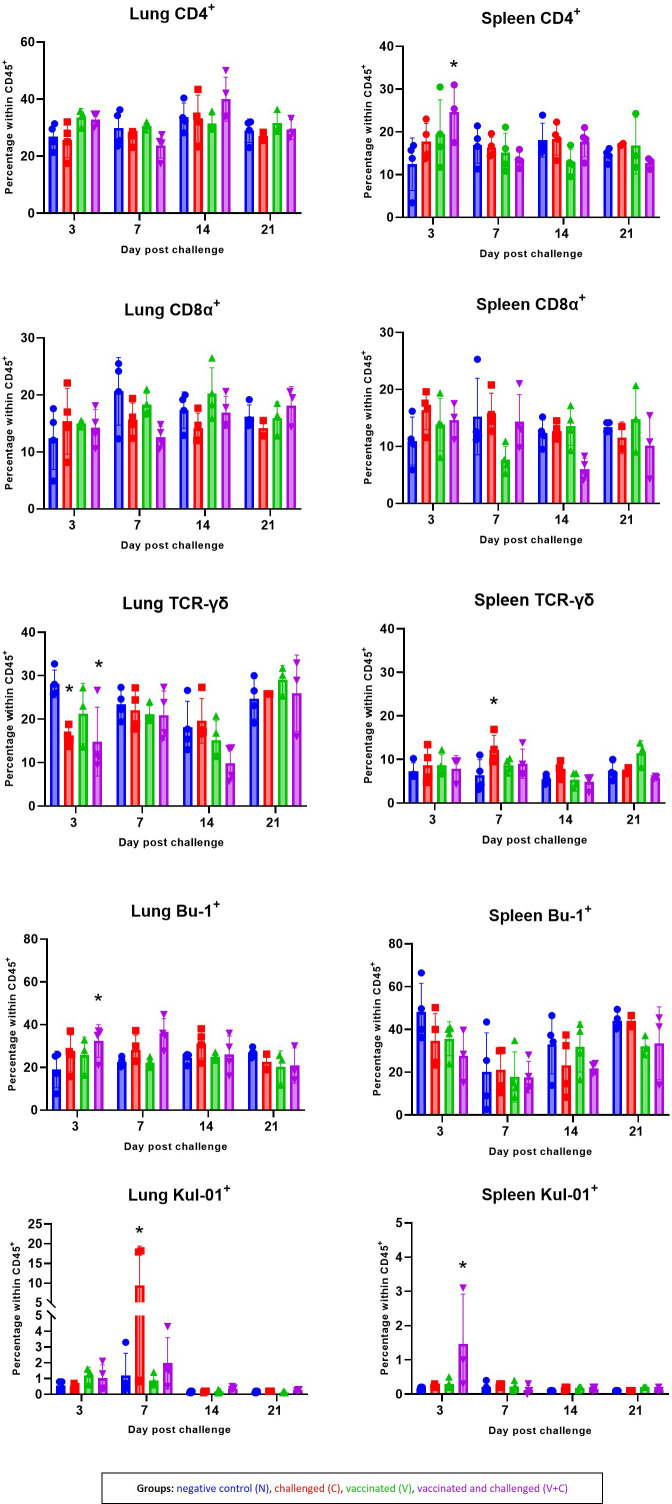
Alterations of CD4^+^, CD8α^+^, TCR-γδ^+^, Bu-1^+^ and Kul-01^+^ cell subpopulations following vaccination and/or challenge analysed by flow cytometry in lung and spleen. The percentage of CD45^+^CD4^+^, CD45^+^CD8α^+^ TCR-γδ^-^, CD45^+^TCR-γδ^+^, CD45^+^CD4^-^CD8α^-^Bu-1^+^ and CD45^+^KUL-01^+^ cells were determined in groups N (blue) C (red), V (green) V+C (purple). Statistical differences were evaluated using linear mixed model in R. Significance was declared at an alpha cut off of 5%. Pairwise comparisons between groups were corrected for multiple testing *via* the Bonferroni-Holm method. Asterisks indicate a significant difference in compare to the control group (*P* ≤ 0.05).

The mean frequencies of CD4, CD8α, TCR-γδ and B cells as well as monocytes/macrophages in the lung and spleen of chickens are shown in **Figure 8**. FCM analyses revealed that the in spleen CD4^+^ cells frequencies significantly increased in the group V+C at 3 dpc (*P*=0.0099). CD8α^+^ cells accounted for the spleen and the lung mononuclear cells, with no significant differences between the groups. Interestingly, in both challenged groups (groups C and V+C), a significant decrease of lung TCR-γδ^+^ cell was detected at 3 dpc compared to control birds (group N) (*P*=0.0222 and 0.0050, respectively). In addition, at 7 dpc an increase was observed in the same T-cell subpopulation in the spleen of challenged birds (*P*=0.0246). For B cells, the only significant variation was limited to an increase in group V+C at 3 dpc in the lung (*P*=0.0461). Lung monocytes/macrophages were elevated in group C at 7 dpc (*P*=0.0029). Additionally, the frequency of those cells in the spleen rose significantly at 3 dpc in vaccinated and challenged birds compared to the control group (*P*=0.00002).

### 3.6 Expression of interferon-γ of re-stimulated mononuclear cells

**Figure 9:**
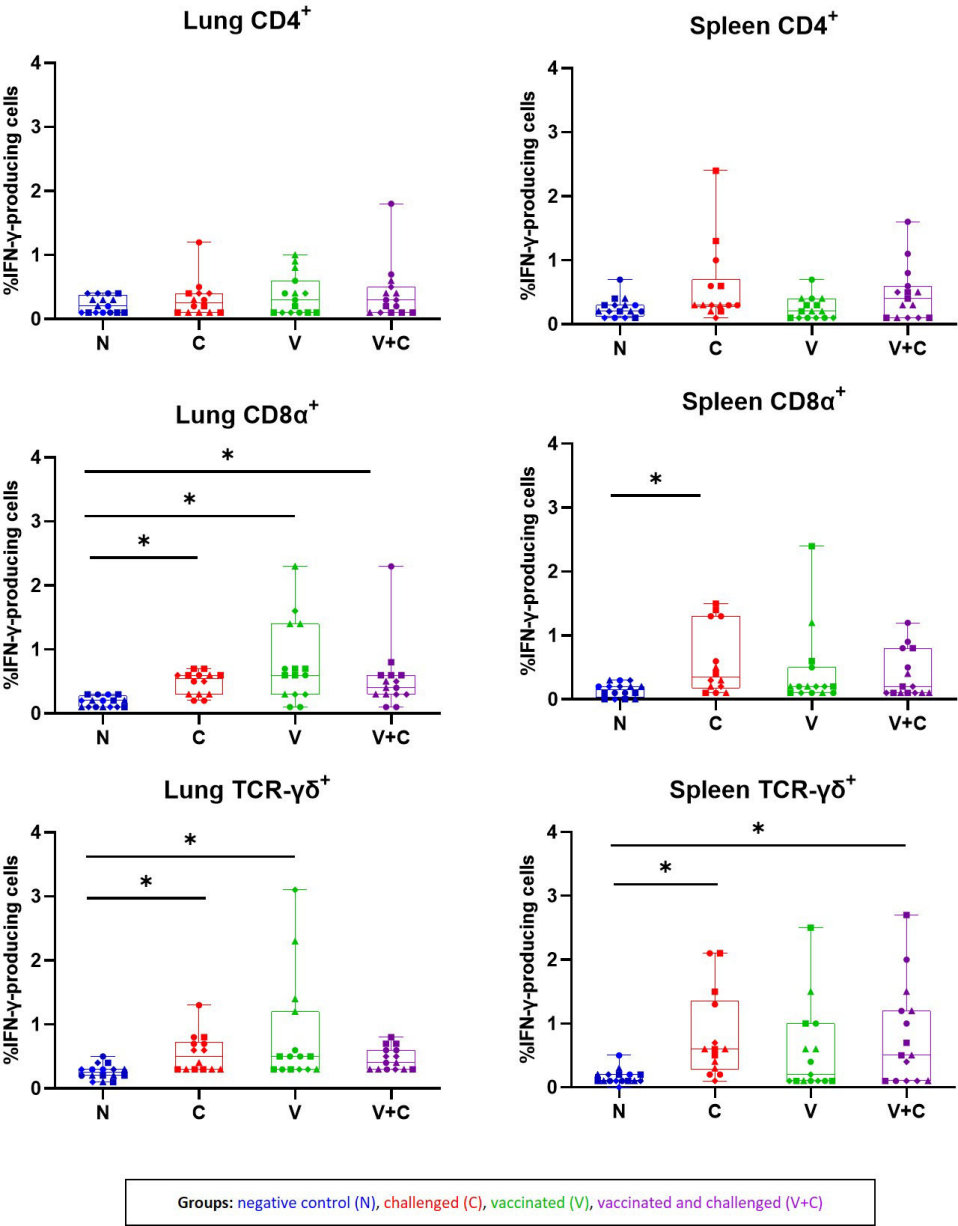
Frequencies of IFN-γ-producing cells subpopulations isolated from lung and spleen of birds following re-stimulation with irradiated APEC. The plots represent the mean frequencies with standard error of IFN-γ-producing CD45^+^CD4^+^, CD45^+^CD8α^+^ TCR-γδ^−^ and CD45^+^TCR-γδ^+^ T cells isolated from lung and spleen following re-stimulation with irradiated APEC. Neither for total Kul-01^+^ nor Bu-1^+^ cells, hardly any IFN-γ producing cells were identified. Statistical differences were evaluated using one-way ANOVA in R. Significance was declared at an alpha cut off of 5%. Pairwise comparisons between groups were corrected for multiple testing *via* the Bonferroni-Holm method. Asterisks indicate a significant difference of the C (red) V (green) V+C (purple) in compare to the N (blue) group (*P* ≤ 0.05). Different symbols represent respective time points: circles: 3 dpc, rectangles: 7 dpc, triangles: 14 dpc and rhombuses: 21 dpc.

*Ex vivo* stimulation of isolated mononuclear cells from chickens with irradiated APEC demonstrated a response of different T-cell subpopulations (CD4^+^, CD8α^+^ and TCR-γδ^+^) by IFN-γ production (**Figure 9**). Statistical analysis showed that results for IFN-γ-producing T-cell subsets did not significantly differ between time points (data not shown). Therefore, we calculated the results of IFN-γ^+^ T cell frequencies from all birds of the respective group together. APEC-specific IFN-γ-producing CD4^+^ T cells were identified by ICS in cells from lung and spleen of birds with no significant changes related to APEC vaccination or challenge. Isolated lung mononuclear cells of the vaccinated and/or challenged birds (groups C, V and V+C) showed a significant increase of IFN-γ^+^CD8α^+^ after re-stimulation with irradiated APEC in comparison to the control birds (*P*=0.0158, 0.0001 and 0.0147, respectively). Additionally, in challenged birds a raise of IFN-γ^+^CD8α^+^ T cells was detected in spleen derived cells (*P*=0.007). APEC-specific IFN-γ^+^TCR-γδ^+^ T cell frequencies from mononuclear cells from lung were higher upon re-stimulation in groups C and V in comparison to control birds (*P*=0.0485 and 0.0009, respectively). For spleen cells, significant differences were determined in groups C and V+C following re-stimulation (*P*=0.0066 and 0.0087, respectively). Monocytes/macrophages did not respond to re-stimulation by IFN-γ production (data not shown).

## 4 Discussion

In the present study, a discriminative vaccination/challenge model of colibacillosis was implemented using commercial pullets. Thereby, we focused on immunological changes caused by irradiated and/or live APEC. The irradiated *E. coli* was administered *via* aerosol route which was found suitable to induce protection in young chickens (5). The used field strain of APEC was previously determined to be associated with colibacillosis and consequently the disease could successfully be reproduced in individual pullets as well. However, the disease could not be reproduced in every non-vaccinated but challenged pullet similar to the outcome of a previous experiment using 2-week-old chickens (5). Nevertheless, clinical signs and typical lesions of colibacillosis such as airsacculitis, perihepatitis and pericarditis, consolidated lungs overlaying fibrinopurulent material and peritonitis were observed in birds of the challenged groups (groups C and V+C).

*E. coli* was isolated from lungs of the majority of birds in groups C and V+C. Accordingly, the lung of challenged birds (groups C and V+C) was the most affected organ and resulted in pathological changes and inflammation, similar to changes in air sac. Macroscopic and microscopic lesions in lung had been suggested to play an important role for entry of APEC into systemic circulation (43). Consequently, systemic bacteremia in challenged birds is most likely the sequel of lesions in lower respiratory organs. Additionally, non-vaccinated but challenged birds (group C) developed slightly more severe lesions in lungs in association with higher organ to body weight ratios compared to other groups. The increase of relative organ weights can be interpreted as an immune response in the lung which was also histopathologically confirmed by the increase of inflammatory cells (44). Neither macroscopic nor microscopic lesions were identified in groups N or V which were not challenged with bacteria; however, *E. coli* was isolated from the lung of some of those birds. This could be explained by the fact that positive re-isolations of *E. coli* from lung of non-infected commercial birds is possible (45). The infection of the spleen is indicative of a systemic infection (33) and in the present work congestion and microscopic changes such as infiltrations of heterophils were observed in this organ in some of the challenged birds (groups C and V+C). In addition, the increase in relative weight of the spleen has formerly been described in colibacillosis (46) which is evident in challenged birds of this experiment as well. Altogether, vaccination reduced necropsy findings and histopathological lesions of the challenge and a lower probability of bacterial re-isolation in group V+C compared to group C, indicating a more effective elimination of bacteria as a result of vaccination. These results supports the protective effect of the non-adjuvanted irradiated APEC vaccine in pullets. Even though, further investigations and optimizations for a clinical protection of pullets against APEC are needed. The composition of the vaccine can be further improved considering optimization in concentration and suitable adjuvants. Furthermore, the vaccination regime and the combination of different kind of vaccines are reported to improve efficacy (47).

Gene expression of cytokines in response to vaccination in combination with APEC challenge has only been described in a few of studies (18, 19, 23) and so far, the effect of irradiated APEC on cytokines has not been investigated at all. Therefore, the cytokines *IL-1β*, *IL-6*, *IL-10* and *IFN-γ* were selected in the present work to identify pro- and anti-inflammatory, regulatory and adaptive pathways activated during vaccination and/or challenge with APEC at different time points after challenge. Consistent with our findings, an upregulation of *IL-1β* in chickens following APEC infection was already shown in the lung and the spleen (17, 19). *IL-1β* is a pro-inflammatory cytokine, which can be synthesized and secreted by a multitude of cell types, with multifunctional attributions and engagement in a variety of host inflammatory and immune responses especially in context of bacterial infections (48, 49). The upregulation of *IL-1β* in spleen samples of vaccinated and/or challenged birds (groups V, C and V+C) is likely because the tissue resident antigen presenting cells in the respiratory tract that first encountered by APEC, will be activated to secrete cytokines and chemokines as a part of the innate immune response (50). This signalling pathway can trigger the attraction of more immune cells like T cells and macrophages to the infected tissue (51). Additionally, the production of cytokines and chemokines can activate systemic immune responses, involving immune organs such as the spleen.

Notably, in our data we observed a remarkable upregulation of *IL-6* mRNA expression only in the lung of vaccinated and/or challenged birds (groups V, C and V+C), which was several folds higher than the expression of other cytokine genes. These findings are also accordant to the fact that the lung was more affected than the spleen showing a higher number of the isolated bacteria, and more macroscopic and microscopic lesions. In consistency with our finding, no increase of *IL-6* was observed in the spleen in a similar study following vaccination with recombinant antigens as vaccine and challenged with APEC (19). This outcome suggests that the application of irradiated or live APEC to the lower respiratory tract causes a local upregulation of *IL-6* which cannot be observed in a systemic expression of this cytokine in the spleen. Functions of *IL-6* as a pro-inflammatory cytokine and its lymphocyte-stimulating role makes it a very useful marker to test immune system activation (16, 49, 52). Generally, the concentration of *IL-6* in the circulation of healthy birds is relatively low but can be rapidly increased in response to foreign stimuli, particularly in disease status or extreme conditions (53). In our experiment, *IL-6* was upregulated, even in vaccinated birds which shows an immune stimulatory effect of irradiated APEC.

Remarkably, birds that received only vaccination showed a similar pattern in upregulation of *IL-1β* and *IL-6* expression in the lung and the spleen compared to challenged birds (groups V+C and C). The explanation might be that irradiated APEC pathogen-associated molecular patterns (PAMPs) such as lipopolysaccharides and flagellin remains intact after irradiation (5, 11). It was reported that these PAMPs can be sensed by specific pattern recognition receptors (54). This recognition can lead to a secretion of pro-inflammatory cytokines like *IL-1β* and *IL-6* as well as chemokines that attract phagocytic heterophils and macrophages to the site of infection as a response. However, this upregulation caused by irradiated APEC was less than the upregulation resulted from live bacteria suggesting less chance of immunopathogenic damage to host tissues (48). Altogether, the ability of irradiated APEC to elicit significant increases in pro-inflammatory cytokines (*IL-1β* and *IL-6*), indicates that the used vaccine activates the respective immune pathways to defend against APEC.

Testing different cytokines’ patterns including Th1 (*IFN-γ*) and regulatory/anti-inflammatory cytokine (*IL-10*) showed no expression differences at different time points except a statistically significant downregulation of *IL-10* at 21 dpc in the lung of birds only challenged (group C). Previously, Ariaans et al. (17) presented an upregulation of cytokines *IFN-γ* and *IL-10* in splenocytes and lung samples one day after inoculation with *E. coli* which dropped, suggesting a short and rapid onset of these cytokines after APEC infection.

Results from the ELISA measuring antibodies (IgY) against APEC in sera from birds did not differ between the groups at any sampling time point before and after challenge. A preceding development against *E. coli* can be suggested according to the housing conditions in a commercial pullet farm. Anyhow, Filho et al. (26) suggested that the protection provided by vaccination with APEC does not necessarily depend on the production of antibodies. In the last mentioned study, the humoral and cell-mediated responses after vaccination with the Poulvac^®^ *E. coli* vaccine, an aroA-deficient mutant strain of APEC serogroup O78, was investigated. Spray vaccination of broiler chicks with the Poulvac^®^ *E. coli* vaccine indicated that after an initial innate immune response, T cell and antibody-mediated protection may be important, as suggested by the number of CD8^+^ cells in the blood and the production of mucosal antibodies that could be seen by the number of circulating CD4^+^TCRVβ1^+^ cells. Importantly, in a recent study investigating the effect of the irradiated vaccine in SPF layer chicks, it was emphasized that the humoral immunity is less important because of the absence of circulating antibodies (IgY) (5). The immunophenotyping analysis of non-stimulated mononuclear cells in the present work revealed phenotypic differences in birds following vaccination and/or challenge with APEC. Analysis of CD4^+^ T cell subset elucidated a significant increase in spleen of vaccinated and challenged birds at 3 dpc. At the same time, the increase of CD4^+^ cells was associated with a rise of macrophages in the spleen. The simultaneous elevation of both cell types can be explained by specific interactions in context of antigen presentation by macrophages to the T-cell subsets which results in the development of adaptive cell-mediated immunity (55). Importantly, it was also found that CD8α^+^ cells remained unchanged during the whole experiment after vaccination and/or challenge in the lung and spleen. In agreement with these results, no significant differences in frequencies of CD8β and CD8α between APEC challenged and control birds were observed in blood mononuclear cells and splenocytes of broilers (17). Changes of γδ T cells were found in relevant numbers in the lung and spleen of pullets in the present work. This T-cell subtype was commonly described as innate-like cell, which has been shown to rapidly elevate in the initial stages of an immune response after a bacterial infection like *Salmonella* Typhimurium (56). In contrast to this, the percentage of TCR-γδ^+^ significantly decreased at 3 dpc in lung mononuclear cells obtained from groups C and V+C following APEC challenge. This finding is not fully understood but Meijerink et al. (24) reported a similar observation in the spleen of broilers infected with *Salmonella* Enteritidis. However, knowledge on the functional role of γδ T cells in immunity to APEC infection in birds is scarce and mechanisms provoked by γδ T cells in protection against this pathogen deserve further elucidation.

Alongside to T cells, we observed a significant increase of B cells (Bu1^+^) at 3 dpc in vaccinated and challenged groups in the lung. It was already shown by bone marrow transcriptome analysis that B cell development is extensively affected by APEC infection in birds (25). This result suggests that vaccination and challenge by APEC induces B cells activation and migration to the site of APEC infection. Moreover, an increase in numbers of B cells was associated with significant upregulation of *IL-6* in the lung of vaccinated and challenged birds. This association might be explained by the function of *IL-6* to induce differentiation of B cells into antibody producing cells which might be important in clinical protection of pullets (48).

Changes of monocytes in blood (17) as well as macrophages in the lung (57) were reported following colibacillosis. Accordingly, in our study the number of Kul-01^+^ cells increased in the lung and the spleen of challenged birds. It was previously described that the number of macrophages in the lung is considered to be important to antagonize APEC (12, 58). The systemic spread of APEC, as observed in the present work, coincided with a significantly increased presence of macrophages in the lung and spleen which suggests macrophages’ role in systemic clearance of the APEC in chickens (17, 57, 59).

The detection of enhanced activation of immune cells by expression of intracellular IFN-γ was shown in several studies investigating vaccine-induced T cell responses in the chicken (60, 61). However, APEC-specific T-cell immune response following vaccination and/or infection of chickens has not been studied. In a previous work, we suggested that the initial T cell responsiveness of naïve mononuclear cells from spleen, lung and blood of chickens to APEC characterized by activation of the IFN-γ and IL-17A signalling pathways is expected to enhance the immune response of host birds which might be important in control of APEC (28). Consequently, it was of interest to investigate whether primed mononuclear cells are capable to recall responses indicating irradiated APEC to be used for vaccination or not. As IFN-γ proofed a key inflammatory biomarker which plays an important and critical role in activation of the cellular immunity (54), the focus of the present study was set only on IFN-γ. *Ex vivo* stimulation of isolated mononuclear cells from pullets with irradiated APEC demonstrated a response of different T-cell subpopulations (CD4^+^, CD8α^+^ and TCR-γδ^+^) by IFN-γ production. Despite the fact that no macroscopic and microscopic lesions have been observed in birds that received vaccination only, re-stimulated lung mononuclear cells showed a significant increase of both IFN-γ^+^CD8α^+^ and IFN-γ^+^TCR-γδ^+^ T cells in comparison to control birds. This suggests that vaccination could prime birds that specifically respond to a challenge by adapted immune cells even though further experiments have to confirm this. Moreover, this result shows that vaccine-induced APEC-specific IFN-γ-producing γδ^+^ and CD8α^+^ T cells contribute to the local immunity in the lung against APEC. Notably, in our experiment isolated lung and spleen mononuclear cells from only challenged pullets showed a significant increase of the same T-cell subpopulations in comparison to control birds whereas vaccinated and challenged chickens showed a significant increase of IFN-γ^+^CD8α^+^ T cells in the lung and IFN-γ^+^TCR-γδ^+^ T cells in the spleen. In general, IFN-γ production seems to play an important role in the immune response against bacteria as it was previously shown that it can subsequently stimulate additional macrophages to clear phagocytosed bacteria (62) or impair intracellular survival by direct killing of infected cells (55). However, we did not observe the production of IFN-γ of chicken macrophages in the present study. Nevertheless, the existence of such cells in avian species should not be denied since they are described in mammals, even in a very low frequency (63). Furthermore, a more precise phenotyping of the IFN-γ-producing cells that can be extended for heterophils and NK cells would be desirable for future investigations. Anyhow, the generated results of the present work elucidated important roles of cell mediated responses against APEC by a clear pathogen-specific T-cell response represented by IFN-γ production.

In conclusion, the present study shows that the application of non-adjuvanted irradiated APEC *via* aerosol route activates the cellular immune response in the target tissue as well as the spleen as systemic immunological organ. The vaccine provides priming of the immune system against invading APEC. Moreover, the results demonstrate the expression of the cytokines *IL-1β* and *IL-6*, cell mediated responses as well as APEC-specific T-cell response dominated by IFN-γ-producing CD8α^+^ and TCR-γδ^+^ subsets to be triggered against colibacillosis.

## Data availability statement

The datasets presented in this study can be found in online repositories. The names of the repository/repositories and accession number(s) can be found in the article/**Supplementary Material**.

## Ethics statement

The trial was approved by the institutional ethics committee and the national authority according to § 8ff of the law for Animal Experiments, Tierversuchsgesetz-TVG (Licence number: BMWF-68.205/0082-V/3b/2019).

## Author contributions

SB, DL, TM and MH conceived and designed the study. SB, KA, SK, SP and DL conducted the animal trial. SB prepared suspensions and VW, GC and RK treated the bacteria with gamma irradiation. CH and KA did bacteriological isolation. SB and TM conducted flow cytometry experiments. SB and SK performed IHC and RT-qPCR. ML did statistical analysis. SB, TM and DL analyzed and interpreted the data. SB drafted the manuscript. DL supervised the study, revised the manuscript critically for essential intellectual content and was responsible for funding acquisition. All authors contributed to the article and approved the submitted version.

## References

[1] Nolan LK Vaillancourt J-P Barbieri NL Logue CM. Colibacillosis. In: Swayne DE Boulianne M Logue CM McDougald LR Nair V Suarez DL, editors. Diseases of poultry, 14th. Hoboken, NJ: John Wiley & Sons, Inc (2019). p. 770–830.

[2] Kathayat D Lokesh D Ranjit S Rajashekara G. Avian pathogenic *Escherichia coli* (APEC): an overview of virulence and pathogenesis factors, zoonotic potential, and control strategies. Pathogens (2021) 10(4):467. doi: 10.3390/pathogens10040467 33921518 PMC8069529

[3] Dziva F Stevens MP. Colibacillosis in poultry: unravelling the molecular basis of virulence of avian pathogenic escherichia coli in their natural hosts. Avian Pathol (2008) 37(4):355–66. doi: 10.1080/03079450802216652 18622850

[4] Sargeant JM Bergevin MD Churchill K Dawkins K Deb B Dunn J. The efficacy of antibiotics to control colibacillosis in broiler poultry: a systematic review. Anim Health Res Rev (2019) 20(2):263–73. doi: 10.1017/S1466252319000264 32081126

[5] Paudel S Hess C Abdelhamid MK Lyrakis M Wijewardana V Kangethe RT. Aerosol delivered irradiated *Escherichia coli* confers serotype-independent protection and prevents colibacillosis in young chickens. Vaccine (2023) 13:1342–53. doi: 10.1016/j.vaccine.2022.12.002 36642629

[6] Ghunaim H Abu-Madi MA Kariyawasam S. Advances in vaccination against avian pathogenic *Escherichia coli* respiratory disease: potentials and limitations. Vet Microbiol (2014) 172(1-2):13–22. doi: 10.1016/j.vetmic.2014.04.019 24878325

[7] Christensen H Bachmeier J Bisgaard M. New strategies to prevent and control avian pathogenic *Escherichia coli* (APEC). Avian Pathol (2021) 50(5):370–81. doi: 10.1080/03079457.2020.1845300 33146543

[8] Viljoen GJ Unger H Wijewardana V Naletoski I. Novel developments and next-generation vaccines. In: Metwally S Viljoen G El Idrissi A, editors. Veterinary vaccines: principles and applications. Hoboken, NJ: John Wiley & Sons (2021). p. 119–34.

[9] Bhatia SS Pillai SD. Ionizing radiation technologies for vaccine development-a mini review. Front Immunol (2022) 13:845514. doi: 10.3389/fimmu.2022.845514 35222438 PMC8873931

[10] Brockstedt DG Bahjat KS Giedlin MA Liu W Leong M Luckett W. Killed but metabolically active microbes: a new vaccine paradigm for eliciting effector T-cell responses and protective immunity. Nat Med (2005) 11(8):853–60. doi: 10.1038/nm1276 16041382

[11] Hieke AS Pillai SD. *Escherichia coli* cells exposed to lethal doses of electron beam irradiation retain their ability to propagate bacteriophages and are metabolically active. Front Microbiol (2018) 9:2138. doi: 10.3389/fmicb.2018.02138 30250460 PMC6139317

[12] Guabiraba R Schouler C. Avian colibacillosis: still many black holes. FEMS Microbiol Lett (2015) 362(15):fnv118. doi: 10.1093/femsle/fnv118 26204893

[13] Alber A Stevens MP Vervelde L. The bird’s immune response to avian pathogenic. Escherichia coli. Avian Pathol (2021) 9:1–0. doi: 10.1080/03079457.2021.1873246 33410704

[14] Sandford EE Orr M Shelby M Li X Zhou H Johnson TJ. Leukocyte transcriptome from chickens infected with avian pathogenic *Escherichia coli* identifies pathways associated with resistance. Results Immunol (2012) 2:44–53. doi: 10.1016/j.rinim.2012.02.003 24371566 PMC3862389

[15] Sun H Bi R Liu P Nolan LK Lamont SJ. Combined analysis of primary lymphoid tissues' transcriptomic response to extra-intestinal *Escherichia coli* (ExPEC) infection. Dev Comp Immunol (2016) 57:99–106. doi: 10.1016/j.dci.2015.12.013 26710679

[16] Alber A Morris KM Bryson KJ Sutton KM Monson MS Chintoan-Uta C. Avian pathogenic *Escherichia coli* (APEC) strain-dependent immunomodulation of respiratory granulocytes and mononuclear phagocytes in CSF1R-reporter transgenic chickens. Front Immunol (2020) 10:3055. doi: 10.3389/fimmu.2019.03055 31998322 PMC6967599

[17] Ariaans MP Matthijs MG van Haarlem D van de Haar P van Eck JH Hensen EJ. The role of phagocytic cells in enhanced susceptibility of broilers to colibacillosis after infectious bronchitis virus infection. Vet Immunol Immunopathol (2008) 123(3-4):240–50. doi: 10.1016/j.vetimm.2008.02.003 PMC7112703 18359518

[18] Sandford EE Orr M Balfanz E Bowerman N Li X Zhou H. Spleen transcriptome response to infection with avian pathogenic *Escherichia coli* in broiler chickens. BMC Genom (2011) 12:469. doi: 10.1186/1471-2164-12-469 PMC3190404 21951686

[19] Van Goor A Stromberg ZR Mellata M. A recombinant multi-antigen vaccine with broad protection potential against avian pathogenic *Escherichia coli* . PloS One (2017) 12(8):e0183929. doi: 10.1371/journal.pone.0183929 28837660 PMC5570496

[20] Peng LY Yuan M Song K Yu JL Li JH Huang JN. Baicalin alleviated APEC-induced acute lung injury in chicken by inhibiting NF-κB pathway activation. Int Immunopharmacol (2019) 72:467–72. doi: 10.1016/j.intimp.2019.04.046 31035089

[21] Goonewardene K Ahmed KA Gunawardana T Popowich S Kurukulasuriya S Karunarathna R. Mucosal delivery of CpG-ODN mimicking bacterial DNA *via* the intrapulmonary route induces systemic antimicrobial immune responses in neonatal chicks. Sci Rep (2020) 10(1):5343. doi: 10.1038/s41598-020-61683-y 32210244 PMC7093454

[22] Soleymani S Tavassoli A Hashemi Tabar G Kalidari GA Dehghani H. Design, development, and evaluation of the efficacy of a nucleic acid-free version of a bacterial ghost candidate vaccine against avian pathogenic *E. coli* (APEC) O78: K80 serotype. Vet Res (2020) 51(1):144. doi: 10.1186/s13567-020-00867-w 33298146 PMC7724879

[23] Elnagar R Elkenany R Younis G. Interleukin gene expression in broiler chickens infected by different *Escherichia coli* serotypes. Vet World (2021) 14(10):2727–34. doi: 10.14202/vetworld.2021.2727-2734 PMC8654741 34903932

[24] Meijerink N Van den Biggelaar RH Van Haarlem DA Stegeman JA Rutten VP Jansen CA. A detailed analysis of innate and adaptive immune responsiveness upon infection with *Salmonella enterica* serotype enteritidis in young broiler chickens. Vet Res (2021) 52(1):109. doi: 10.1186/s13567-021-00978-y 34404469 PMC8369617

[25] Sun H Liu P Nolan LK Lamont SJ. Avian pathogenic *Escherichia coli* (APEC) infection alters bone marrow transcriptome in chickens. BMC Genom (2015) 16(1):690. doi: 10.1186/s12864-015-1850-4 PMC4570614 26369556

[26] Filho TF Fávaro C Ingberman M Beirão BC Inoue A Gomes L. Effect of spray *Escherichia coli* vaccine on the immunity of poultry. Avian Dis (2013) 57(3):671–6. doi: 10.1637/10456-112612-ResNote.1 24283136

[27] Gharib AA Hamouda AM Bdel-Wahab AA Fawzy M. Protective efficacy of a commercial live attenuated *aroA* mutant vaccine against avian pathogenic *Escherichia coli* challenge in broilers. Zagazig Vet J (2017) 45(4):366–75. doi: 10.21608/ZVJZ.2017.7867

[28] Bagheri S Paudel S Wijewardana V Kangethe RT Cattoli G Hess M. Production of interferon gamma and interleukin 17A in chicken T-cell subpopulations hallmarks the stimulation with live, irradiated and killed avian pathogenic. Escherichia coli. Dev Comp Immunol (2022) 133:104408. doi: 10.1016/j.dci.2022.104408 35390358

[29] Zloch A Kuchling S Hess M Hess C. Influence of alternative husbandry systems on postmortem findings and prevalence of important bacteria and parasites in layers monitored from end of rearing until slaughter. Vet Rec. (2018) 182(12):350. doi: 10.1136/vr.104632 29434034

[30] Rezaee MS Liebhart D Hess C Hess M Paudel S. Bacterial infection in chicken embryos and consequences of yolk sac constitution for embryo survival. Vet Pathol (2021) 58(1):71–9. doi: 10.1177/0300985820960127 33016240

[31] Paudel S Fink D Abdelhamid MK Zöggeler A Liebhart D Hess M. Aerosol is the optimal route of respiratory tract infection to induce pathological lesions of colibacillosis by a lux-tagged avian pathogenic *Escherichia coli* in chickens. Avian Pathol (2021) 1:1–0. doi: 10.1080/03079457.2021.1978392 34505551

[32] Landman WJM Buter GJ Dijkman R van Eck JHH. *In vivo* typing of escherichia coli obtained from laying chickens with the *E. coli* peritonitis syndrome. Avian Pathol (2021) 50(5):436–46. doi: 10.1080/03079457.2021.1962004 34351217

[33] Antao EM Glodde S Li G Sharifi R Homeier T Laturnus C. The chicken as a natural model for extraintestinal infections caused by avian pathogenic *Escherichia coli* (APEC). Microb Pathog (2008) 45(5-6):361–9. doi: 10.1016/j.micpath.2008.08.005 18848980

[34] Abdelhamid MK Rychlik I Hess C Hatfaludi T Crhanova M Karasova D. Typhlitis induced by *Histomonas meleagridis* affects relative but not the absolute *Escherichia coli* counts and invasion in the gut in turkeys. Vet Res (2021) 52(1):92. doi: 10.1186/s13567-021-00962-6 34158121 PMC8220719

[35] Mitra T Bilic I Hess M Liebhart D. The 60S ribosomal protein L13 is the most preferable reference gene to investigate gene expression in selected organs from turkeys and chickens, in context of different infection models. Vet Res (2016) 47(1):1–9. doi: 10.1186/s13567-016-0388-z 27765062 PMC5073923

[36] Powell FL Rothwell L Clarkson MJ Kaiser P. The turkey, compared to the chicken, fails to mount an effective early immune response to *Histomonas meleagridis* in the gut. Parasite Immunol (2009) 31(6):312–27. doi: 10.1111/j.1365-3024.2009.01113.x 19493211

[37] Bustin SA Benes V Garson JA Hellemans J Huggett J Kubista M. The MIQE guidelines: minimum information for publication of quantitative real-time PCR experiments. Clin Chem (2009) 55(4):611–22. doi: 10.1373/clinchem.2008.112797 19246619

[38] Livak KJ Schmittgen TD. Analysis of relative gene expression data using real-time quantitative PCR and the 2– ΔΔCT. Methods (2001) 25(4):402–8. doi: 10.1006/meth.2001.1262 11846609

[39] R-Core-Team. A language and environment for statistical computing’ (2022). Vienna, Austria: R Foundation for Statistical Computing. Available at: https://www.r-project.org/.

[40] Lenth R Singmann H Love J Buerkner P Herve M. Emmeans: estimated marginal means, aka least-squares means (2022). Available at: https://cran.r-project.org/package=emmeans.

[41] Bates D Mächler M Bolker B Walker S. Fitting linear mixed-effects models using lme4. J Stat Software (2015) 67(1):1–48. doi: 10.18637/jss.v067.i01

[42] Fox J Weisberg S. An r companion to applied regression. 3rd ed. Thousand Oaks CA: Sage (2019). Available at: https://socialsciences.mcmaster.ca/jfox/Books.

[43] Pourbakhsh SA Boulianne M Martineau-Doize B Fairbrother JM. Virulence mechanisms of avian fimbriated *Escherichia coli* in experimentally inoculated chickens. Vet Microbiol (1997) 58(2-4):195–213. doi: 10.1016/s0378-1135(97)00163-6 9453131

[44] Kromann S Olsen RH Bojesen AM Jensen HE Thøfner I. Protective potential of an autogenous vaccine in an aerogenous model of *Escherichia coli* infection in broiler breeders. Vaccines (2021) 9:1233. doi: 10.3390/vaccines9111233 34835164 PMC8624668

[45] Kromann S Olsen RH Bojesen AM Jensen HE Thøfner I. Development of an aerogenous *Escherichia coli* infection model in adult broiler breeders. Sci Rep (2021) 11(1):19556. doi: 10.1038/s41598-021-98270-8 34599201 PMC8486767

[46] Huff WE Huff GR Rath NC Balog JM Donoghue AM. Evaluation of aerosol spray and intramuscular injection of bacteriophage to treat an *Escherichia coli* respiratory infection. Poult Sci (2003) 82(7):1108–12. doi: 10.1093/ps/82.7.1108 12872966

[47] Koutsianos D Gantelet H Franzo G Lecoupeur M Thibault E Cecchinato M. An assessment of the level of protection against colibacillosis conferred by several autogenous and/or commercial vaccination programs in conventional pullets upon experimental challenge. Veterinary Sci (2020) 7:80. doi: 10.3390/vetsci7030080 PMC7559755 32629910

[48] Bean AGD Lowenthal JW. Chapter 9 - avian cytokines and their receptors. In: Kaspers B Schat KA Göbel TW Vervelde L, editors. Avian immunology, 3rd ed. Boston: Academic Press (2022). p. 249–76. doi: 10.1016/B978-0-12-818708-1.00024-5

[49] Lu M Lee Y Lillehoj HS. Evolution of developmental and comparative immunology in poultry: the regulators and the regulated. Dev Comp Immunol (2022) 138:104525. doi: 10.1016/j.dci.2022.104525 36058383

[50] Barker RN Erwig LP Hill KS Devine A Pearce WP Rees AJ. Antigen presentation by macrophages is enhanced by the uptake of necrotic, but not apoptotic, cells. Clin Exp Immunol (2002) 127(2):220–5. doi: 10.1046/j.1365-2249.2002.01774.x PMC1906351 11876743

[51] Smith AL Fiddaman SR. Chapter 8.6 - pattern recognition receptors. In: Kaspers B Schat KA Göbel TW Vervelde L, editors. Avian immunology, 3rd ed. Boston: Academic Press (2022). p. 231–48. doi: 10.1016/B978-0-12-818708-1.00026-9

[52] Weerts EA Matthijs MG Bonhof J van Haarlem DA Dwars RM Gröne A. The contribution of the immune response to enhanced colibacillosis upon preceding viral respiratory infection in broiler chicken in a dual infection model. Vet Immunol Immunopathol (2021) 238:110276. doi: 10.1016/j.vetimm.2021.110276 34126552

[53] Hunter CA Jones SA. IL-6 as a keystone cytokine in health and disease. Nat Immunol (2015) 16(5):448–57. doi: 10.1038/ni.3153 25898198

[54] Wigley P. The immune system of the chicken. poultry health: a guide for professionals, CABI books. Wallingford, UK: CABI International (2021) p. 8–19. doi: 10.1079/9781789245042.0002

[55] Sutton K Balic A Kaspers B Vervelde L. Chapter 8.1 - macrophages and dendritic cells. In: Kaspers B Schat KA Göbel TW Vervelde L, editors. Avian immunology, 3rd ed. Boston: Academic Press (2022). p. 167–95. doi: 10.1016/B978-0-12-818708-1.00003-8

[56] Pieper J Methner U Berndt A. Characterization of avian γδ T-cell subsets after *Salmonella enterica* serovar typhimurium infection of chicks. Infect Immun (2011) 79(2):822–9. doi: 10.1128/IAI.00788-10 PMC3028855 21078853

[57] Matthijs MG Ariaans MP Dwars RM van Eck JH Bouma A Stegeman A. Course of infection and immune responses in the respiratory tract of IBV infected broilers after superinfection with e. coli. Vet Immunol Immunopathol (2009) 127(1-2):77–84. doi: 10.1016/j.vetimm.2008.09.016 18976820

[58] Mellata M Dho-Moulin M Dozois CM Curtiss IIIR Lehoux B Fairbrother JM. Role of avian pathogenic *Escherichia coli* virulence factors in bacterial interaction with chicken heterophils and macrophages. Infect Immun (2003) 71(1):494–503. doi: 10.1128/IAI.71.1.494-503.2003 12496200 PMC143357

[59] Yu K Gu MJ Pyung YJ Song KD Park TS Han SH. Characterization of splenic MRC1hiMHCIIlo and MRC1loMHCIIhi cells from the monocyte/macrophage lineage of white leghorn chickens. Vet Res (2020) 51(1):73. doi: 10.1186/s13567-020-00795-9 32460863 PMC7251834

[60] Dalgaard TS Norup LR Juul-Madsen HR. Detection of avian antigen-specific T cells induced by viral vaccines. In: Thomas S, editor. Vaccine design: methods and protocols, volume 2: vaccines for veterinary diseases. New York, NY: Springer New York (2016). p. 77–88. doi: 10.1007/978-1-4939-3389-1_5 27076291

[61] Lagler J Schmidt S Mitra T Stadler M Wernsdorf P Grafl B. Comparative investigation of IFN-γ-producing T cells in chickens and turkeys following vaccination and infection with the extracellular parasite *Histomonas meleagridis* . Dev Comp Immunol (2021) 116:103949. doi: 10.1016/j.dci.2020.103949 33253751

[62] Peng L Matthijs MG Haagsman HP Veldhuizen EJ. Avian pathogenic *Escherichia coli*-induced activation of chicken macrophage HD11 cells. Dev Comp Immunol (2018) 87:75–83. doi: 10.1016/j.dci.2018.05.019 29890365

[63] Robinson CM O’Dee D Hamilton T Nau GJ. Cytokines involved in interferon-γ production by human macrophages. J Innate Immun (2010) 2(1):56–65. doi: 10.1159/000247156 20375623 PMC2943519

