# Supplementary material for: Aerosol vaccination of chicken pullets with irradiated avian pathogenic Escherichia coli induces a local immunostimulatory effect
Source: Front Immunol. 2023 May 16;14:1185232. doi: 10.3389/fimmu.2023.1185232 (PMC10227613; doi:10.3389/fimmu.2023.1185232)
Supplement: Supplementary file 1 [file DataSheet_1.docx]

Supplementary Material

Aerosol vaccination of chicken pullets with irradiated avian pathogenic *Escherichia coli* induces a local immunostimulatory effect

Sina Bagheri, Taniya Mitra, Surya Paudel, Kamal Abdelhamid, Simon Könnyü, Viskam Wijewardana, Richard Thiga Kangethe, Giovanni Cattoli, Manolis Lyrakis, Claudia Hess, Michael Hess, Dieter Liebhart^*^

*** Correspondence:** Dieter Liebhart: dieter.liebhart@vetmeduni.ac.at

# Supplementary Tables

**Supplementary Table 1**. Lesion-scoring scheme.

| Organ | Score | Lesion |
| --- | --- | --- |
| thoracic air sacs | 0 | no pathological changes |
|  | 1 | slight opaque and/or thickened membranes |
|  | 2 | moderate to severe thickened air sac with fibrin |
| heart | 0 | no pathological changes |
|  | 1 | opacity with lack of transparency of pericardium |
|  | 2 | thickened pericardium with marked pericarditis |
| liver | 0 | no pathological changes |
|  | 1 | slight amount of fibrin in capsule |
|  | 2 | marked perihepatitis with massive amount of fibrin |
| lung | 0 | no pathological changes |
|  | 1 | lesions are ≤ 1/2 of the of the lung (uni- or bilateral) |
|  | 2 | lesions are ≥ 1/2 of the of the lung (uni- or bilateral) |
| spleen | 0 | no pathological changes |
|  | 1 | congestion |
| peritoneum | 0 | no pathological changes |
|  | 1 | slight edematous exudates and/or fibrin spots |
|  | 2 | marked edematous with fibrinous masses |

**Supplementary Table 2.** Reverse transcription-qPCR primers and probes. 100nM of probe concentrations was used for all genes.

| **Target** | **Primer and probe sequences** | **Primer concentration (nM)** | **Accession number** | **Reference** |
| --- | --- | --- | --- | --- |
| *IFN-γ* | F: GTGAAGAAGGTGAAAGATATCATGGA  R: GCTTTGCGCTGGATTCTCA  P: HEX-TGGCCAAGCTCCCGATGAACGA | 600 | NM_205149.1 | 36 |
| *IL-10* | F: CATGCTGCTGGGCCTGAA  R: CGTCTCCTTGATCTGCTTGATG  P: ROX-CGACGATGCGGCGCTGTCA | 400 | NM_001004414.4 | 36 |
| *IL-6* | F: GCTCGCCGGCTTCGA  R: GGTAGGTCTGAAAGGCGAACAG  P: HEX-AGGAGAAATGCCTGACGAAGCTCTCCA | 600 | NM_204628.2 | 36 |
| *IL-1β* | F: GCTCTACATGTCGTGTGTGATGAG  R: TGTCGATGTCCCGCATGA  P: CY5-CCACACTGCAGCTGGAGGAAGCC | 400 | XM_046931582.1 | 36 |
| *TBP* | F: CATTCCAGGTGCGTGAACTC  R: TTAAGCCTGGTGCTGGATCA  P: ROX-TCCTCGTCCTCCGCCGCGAG | 700 | NM_205103.1 | 35 |
| *TFRC* | F: CATTCCAGGTGCGTGAACTC  R: TTAAGCCTGGTGCTGGATCA  P: FAM -TCCTCGTCCTCCGCCGCGAG | 400 | NM_205256.2 | 35 |

**Supplementary Table 3**. The Cq values of expressed genes in lung and spleen of each bird. The expression levels of each gene were quantified at least two times and presented as an average.

This data is available online via the link https://phaidra.vetmeduni.ac.at/o:1401.

| **Bird**  **identification** | **Cq value** | | | | | | | | | | | |
| --- | --- | --- | --- | --- | --- | --- | --- | --- | --- | --- | --- | --- |
|  | **Lung IL-1b** | **Lung IL-6** | **Lung IL-10** | **Lung IFN-γ** | **Lung TBP** | **Lung TFRC** | **Spleen IL-1b** | **Spleen IL-6** | **Spleen IL-10** | **Spleen IFN-**γ | **Spleen TFRC** | **Spleen TBP** |
| **1** | 25.62 | 26.53 | 27.81 | 32.21 | 25.22 | 25.61 | 27.92 | 28.84 | 30.33 | 27.28 | 23.35 | 22.68 |
| **2** | 26.63 | 29.71 | 29.13 | 32.72 | 24.36 | 24.37 | 26.67 | 26.20 | 29.87 | 28.99 | 23.75 | 23.45 |
| **3** | 26.67 | 27.87 | 25.92 | 29.90 | 23.84 | 23.25 | 26.72 | 25.65 | 26.37 | 26.92 | 22.86 | 22.60 |
| **4** | 27.46 | 29.16 | 26.86 | 28.74 | 23.45 | 22.64 | 27.36 | 24.23 | 30.32 | 28.66 | 22.59 | 22.99 |
| **5** | 28.36 | 31.31 | 27.99 | n.a.^a^ | 25.47 | 25.64 | 25.83 | 27.47 | 26.70 | 26.76 | 21.56 | 21.66 |
| **6** | 25.29 | 27.55 | 26.88 | 31.84 | 24.82 | 25.15 | 29.21 | 26.79 | 29.96 | 27.84 | 23.60 | 23.26 |
| **7** | 30.14 | 32.12 | n.a. | n.a. | 28.57 | 29.09 | 25.60 | 25.05 | 27.60 | 27.39 | 22.18 | 22.25 |
| **8** | 26.29 | 26.62 | 27.57 | 31.00 | 23.28 | 23.28 | 28.19 | 28.11 | 27.98 | 27.10 | 22.35 | 22.51 |
| **9** | 27.25 | 29.60 | 27.76 | 33.78 | 25.84 | 26.21 | 26.09 | 27.54 | 27.84 | 27.14 | 21.79 | 21.53 |
| **10** | 26.68 | 26.60 | 26.31 | 31.15 | 24.17 | 23.73 | 26.91 | 27.16 | 26.08 | 26.38 | 21.94 | 21.41 |
| **11** | 26.37 | 28.61 | 25.65 | 31.38 | 24.46 | 23.70 | 27.14 | 25.76 | 26.23 | 26.27 | 22.36 | 22.03 |
| **12** | 26.14 | 27.88 | 26.14 | 32.39 | 24.57 | 25.38 | 26.34 | 28.22 | 26.95 | 26.76 | 21.76 | 21.71 |
| **13** | 25.37 | 25.38 | 26.03 | 31.57 | 24.73 | 24.95 | 27.02 | 24.60 | 26.06 | 26.98 | 22.15 | 21.81 |
| **14** | 26.93 | 28.68 | 26.60 | 32.33 | 24.53 | 24.68 | 25.76 | 27.82 | 25.77 | 26.62 | 22.12 | 21.80 |
| **15** | 27.60 | 29.23 | 26.30 | 32.68 | 25.73 | 25.37 | 27.73 | 25.26 | 27.09 | 26.15 | 21.85 | 21.50 |
| **16** | 26.90 | 29.56 | 26.16 | 32.09 | 24.60 | 24.55 | 27.42 | 28.04 | 26.80 | 27.35 | 22.19 | 21.89 |
| **17** | 22.51 | 22.85 | 26.24 | 29.08 | 24.05 | 23.55 | 24.56 | 23.33 | 28.36 | 27.13 | 22.83 | 23.03 |
| **18** | 21.86 | 21.70 | 25.37 | 27.44 | 23.18 | 22.44 | 23.42 | 24.47 | 26.81 | 27.25 | 22.81 | 22.97 |
| **20** | 23.46 | 22.35 | 26.38 | 29.27 | 25.80 | 24.34 | 22.70 | 22.33 | 27.98 | 27.16 | 22.62 | 23.39 |
| **21** | 23.57 | 24.83 | 27.29 | 33.20 | 25.08 | 24.60 | 23.88 | 25.56 | 32.19 | 25.64 | 20.87 | 20.84 |
| **22** | 27.12 | 29.62 | 28.53 | 32.29 | 25.11 | 23.79 | 27.48 | 23.96 | 29.50 | 26.97 | 21.76 | 22.07 |
| **24** | 26.75 | 28.96 | 28.56 | 31.31 | 23.40 | 21.79 | 25.55 | 27.03 | 29.08 | 26.99 | 21.18 | 21.78 |
| **25** | 24.60 | 24.35 | 28.61 | 29.92 | 22.99 | 22.16 | 24.02 | 25.99 | 27.63 | 26.42 | 21.11 | 21.36 |
| **26** | 26.72 | 28.62 | 27.24 | 30.69 | 23.79 | 23.27 | 26.69 | 27.44 | 27.26 | 26.98 | 22.06 | 22.05 |
| **27** | 25.94 | 26.97 | 26.19 | 30.02 | 22.94 | 21.91 | 25.12 | 26.65 | 27.08 | 25.60 | 20.66 | 21.27 |
| **28** | 24.82 | 25.32 | 25.77 | 30.08 | 23.66 | 23.37 | 26.93 | 26.22 | 26.45 | 25.63 | 21.80 | 21.95 |
| **29** | 26.35 | 28.66 | 27.23 | 30.05 | 22.87 | 22.36 | 26.84 | 27.58 | 28.85 | 26.38 | 21.80 | 21.90 |
| **30** | 27.10 | 29.58 | 29.58 | 33.79 | 25.98 | 25.42 | 28.08 | 27.47 | 31.12 | 28.01 | 22.64 | 22.88 |
| **31** | n.a. | 25.28 | 32.25 | n.a. | 25.67 | 23.61 | 27.59 | 25.56 | 28.61 | 27.25 | 21.90 | 22.41 |
| **32** | 26.59 | 28.50 | 27.30 | 31.34 | 23.96 | 23.89 | 27.72 | 27.58 | 27.02 | 26.84 | 22.00 | 21.94 |
| **33** | 23.62 | 24.27 | 26.58 | 33.22 | 26.06 | 25.56 | 24.86 | 24.33 | 27.24 | 25.98 | 21.87 | 21.94 |
| **34** | 23.38 | 24.00 | 27.99 | 34.59 | 26.24 | 26.12 | 22.60 | 26.82 | 26.31 | 27.93 | 22.14 | 22.39 |
| **35** | 23.78 | 25.68 | 26.68 | 30.63 | 22.90 | 22.97 | 23.25 | 24.44 | 25.29 | 26.42 | 22.15 | 21.90 |
| **36** | 23.80 | 24.50 | 28.49 | 32.52 | 24.28 | 23.97 | 23.90 | 27.05 | 27.57 | 26.89 | 22.56 | 22.34 |
| **37** | 26.02 | 28.80 | 27.07 | 31.95 | 24.29 | 24.10 | 26.10 | 27.84 | 26.80 | 26.41 | 21.66 | 21.96 |
| **38** | 26.31 | 28.38 | 27.76 | 34.00 | 24.83 | 24.12 | 26.23 | 24.88 | 26.57 | 27.60 | 22.35 | 22.20 |
| **39** | 25.01 | 28.35 | 26.46 | 32.67 | 25.55 | 25.66 | 25.77 | 27.23 | 27.28 | 26.49 | 21.28 | 21.41 |
| **40** | 27.41 | 30.61 | 30.14 | 33.69 | 26.30 | 25.52 | 25.61 | 27.45 | 28.29 | 28.33 | 23.57 | 23.48 |
| **41** | 25.89 | 25.61 | 25.51 | 31.72 | 26.10 | 25.42 | 27.98 | 25.40 | 27.76 | 27.09 | 21.55 | 21.99 |
| **42** | 26.61 | 28.48 | 27.51 | 31.72 | 23.95 | 22.95 | 25.92 | 27.73 | 27.30 | 26.88 | 21.49 | 21.36 |
| **43** | 25.35 | 27.61 | 25.52 | 32.04 | 24.93 | 24.40 | 25.10 | 25.78 | 25.46 | 26.55 | 21.93 | 22.13 |
| **45** | 27.46 | 29.44 | 27.48 | 34.30 | 25.44 | 24.52 | 26.60 | 26.88 | 27.47 | 27.75 | 21.84 | 21.99 |
| **46** | 27.28 | 24.14 | 27.58 | 34.82 | 26.56 | 25.72 | 27.65 | 24.93 | 27.71 | 27.59 | 22.53 | 22.30 |
| **47** | 27.04 | 25.23 | 27.74 | 34.65 | 26.24 | 26.31 | 26.58 | 26.42 | 26.73 | 26.31 | 22.01 | 21.91 |
| **48** | 25.87 | 27.17 | 26.33 | 31.07 | 24.03 | 23.30 | 26.47 | 26.76 | 26.60 | 28.11 | 23.20 | 23.11 |
| **49** | 22.84 | 25.72 | 26.92 | 32.12 | 26.15 | 24.92 | 21.95 | 24.61 | 26.48 | 26.20 | 22.07 | 22.30 |
| **50** | 24.01 | 24.71 | 28.21 | 32.32 | 26.86 | 25.29 | 23.50 | 23.59 | 27.97 | 29.66 | 22.41 | 23.18 |
| **51** | 23.47 | 24.60 | 27.96 | 30.41 | 24.51 | 23.58 | 22.81 | 23.54 | 25.64 | 25.66 | 21.46 | 22.84 |
| **52** | 22.17 | 23.53 | 28.36 | 34.50 | 27.30 | 25.62 | 22.34 | 25.73 | 27.55 | 27.62 | 22.27 | 23.84 |
| **53** | 25.02 | 25.73 | 26.13 | 30.84 | 27.32 | 27.22 | 26.97 | 27.70 | 27.82 | 26.10 | 21.06 | 22.27 |
| **54** | 25.43 | 27.04 | 26.11 | 30.82 | 25.23 | 24.79 | 27.69 | 25.52 | 26.75 | 28.03 | 23.29 | 23.54 |
| **55** | 27.42 | 28.14 | 28.23 | 31.72 | 26.26 | 25.47 | 27.33 | 29.92 | 28.46 | 24.48 | 20.13 | 21.45 |
| **56** | 24.09 | 27.39 | 26.58 | 30.17 | 23.73 | 22.19 | 26.23 | 29.06 | 26.24 | 27.14 | 21.33 | 22.72 |
| **57** | 26.37 | 24.16 | 25.84 | 33.57 | 27.08 | 25.47 | 29.00 | 26.68 | 27.53 | 26.58 | 21.62 | 22.51 |
| **58** | 28.45 | 30.04 | 30.02 | 32.29 | 25.50 | 24.51 | 27.84 | 28.53 | 27.89 | 26.92 | 22.21 | 22.80 |
| **59** | n.a. | n.a. | n.a. | n.a. | n.a. | n.a. | 26.67 | 26.44 | 27.03 | 27.30 | 21.18 | 22.08 |
| **60** | 26.94 | 27.71 | 27.17 | 30.72 | 24.41 | 22.93 | 27.58 | 27.65 | 27.60 | 27.56 | 22.64 | 23.13 |
| **61** | 26.94 | 27.62 | 26.98 | 32.96 | 26.04 | 25.28 | 27.68 | 29.37 | 26.24 | 25.31 | 21.00 | 21.77 |
| **62** | 25.68 | 29.28 | 30.89 | 34.88 | 27.04 | 25.18 | 27.42 | 25.74 | 29.77 | 27.67 | 23.02 | 23.13 |
| **63** | 27.72 | 28.17 | 27.64 | 34.42 | 26.76 | 25.46 | 27.72 | 27.30 | 26.11 | 26.45 | 21.90 | 22.23 |

^a^ n.a.: not available

**Supplementary Table 4.** Antibody panels used to determine immune cell populations of chickens by flow cytometry. Antibody panel (a) was used for phenotyping of directly isolated cells from lung and spleen. Mononuclear cells from the same organs have been re-stimulated with irradiated APEC before staining with antibody panel (b) was applied.

| Antibody panel | Antigen | Clone | Isotype | Fluorochrome | Labelling strategy | Source of primary Ab |
| --- | --- | --- | --- | --- | --- | --- |
| (a) | CD45 | LT40 | IgM | APC | directly conjugated | Southern-Biotech |
|  | CD8α | 3-298 | IgG2bκ | BV421 | secondary antibody^1^ | Southern-Biotech |
|  | CD4 | CT4 | IgG1κ | PE-Cy7 | directly conjugated | Southern-Biotech |
|  | TCR-γδ | TCR-1 | IgG1κ | FITC | directly conjugated | Southern-Biotech |
|  | Bu-1 | AV20 | IgG1κ | BV510 | biotin-streptavidin^2^ | Southern-Biotech |
|  | macrophage/monocyte | Kul-01 | IgG1κ | PE | directly conjugated | Southern-Biotech |
|  |  |  |  |  |  |  |
| (b) | CD45 | LT40 | IgM | APC | directly conjugated | Southern-Biotech |
|  | CD8α | 3-298 | IgG2bκ | BV421 | secondary antibody | Southern-Biotech |
|  | CD4 | CT4 | IgG1κ | PE-Cy7 | directly conjugated | Southern-Biotech |
|  | TCR-γδ | TCR-1 | IgG1κ | BV510 | biotin-streptavidin^2^ | Southern-Biotech |
|  | macrophage/monocyte | Kul-01 | IgG1κ | FITC | directly conjugated | Southern-Biotech |
|  | IFN-γ | 12F7 | IgG2a | PE | secondary antibody^3^ | provided^4^ |

^1^ Goat anti-mouse IgG2b-BV421, Jackson Immuno Research

^2^ Brilliant Violet 510^TM^ Streptavidin, BioLegend

^3^ Goat anti-mouse IgG2a-Fab RPE, Southern Biotech

^4^ Kindly provided by Dr. Fabienne Rauw, Sciensano

**Supplementary Table 5.** Lesion scoring and bacteria detection in air sac, liver, heart and peritoneum of birds.

| **Group** | **Bird identification** | **Day post challenge (dpc)** |  | **Lesion score** | | | |  | **Bacterial detection** | | |
| --- | --- | --- | --- | --- | --- | --- | --- | --- | --- | --- | --- |
|  |  |  |  | **Air sac** | **Liver** | **Heart** | **Peritoneum** |  | **Air sac** | **Liver** | **Heart** |
| **Negative control** | 1 | 3 |  | 0 | 0 | 0 | 0 |  | - | + | - |
|  | 2 | 3 |  | 0 | 0 | 0 | 0 |  | - | - | - |
|  | 3 | 3 |  | 0 | 0 | 0 | 0 |  | - | - | - |
|  | 4 | 3 |  | 0 | 0 | 0 | 0 |  | - | - | - |
|  | 5 | 7 |  | 0 | 0 | 0 | 0 |  | - | - | - |
|  | 6 | 7 |  | 0 | 0 | 0 | 0 |  | - | - | - |
|  | 7 | 7 |  | 0 | 0 | 0 | 0 |  | - | - | - |
|  | 8 | 7 |  | 0 | 0 | 0 | 0 |  | - | + | - |
|  | 9 | 14 |  | 0 | 0 | 0 | 0 |  | - | - | - |
|  | 10 | 14 |  | 0 | 0 | 0 | 0 |  | - | - | - |
|  | 11 | 14 |  | 0 | 0 | 0 | 0 |  | - | - | - |
|  | 12 | 14 |  | 0 | 0 | 0 | 0 |  | - | - | - |
|  | 13 | 21 |  | 0 | 0 | 0 | 0 |  | - | - | - |
|  | 14 | 21 |  | 0 | 0 | 0 | 0 |  | - | - | - |
|  | 15 | 21 |  | 0 | 0 | 0 | 0 |  | - | - | - |
|  | 16 | 21 |  | 0 | 0 | 0 | 0 |  | - | + | - |
| **Challenged** | 17 | 3 |  | 2 | 1 | 2 | 2 |  | - | - | - |
|  | 18 | 3 |  | 2 | 1 | 2 | 2 |  | - | - | - |
|  | 19 **^a^** | 3 |  | 2 | 2 | 2 | 2 |  | + | - | + |
|  | 20 | 3 |  | 1 | 1 | 1 | 2 |  | + | + | - |
|  | 21 | 3 |  | 2 | 1 | 1 | 2 |  | - | + | - |
|  | 22 | 7 |  | 0 | 0 | 0 | 0 |  | - | + | - |
|  | 23 **^a^** | 3 |  | 2 | 2 | 2 | 2 |  | + | - | + |
|  | 24 | 7 |  | 0 | 0 | 0 | 0 |  | - | - | - |
|  | 25 | 7 |  | 1 | 1 | 1 | 1 |  | - | - | - |
|  | 26 | 7 |  | 0 | 0 | 0 | 1 |  | - | - | - |
|  | 27 | 14 |  | 0 | 0 | 0 | 0 |  | - | - | - |
|  | 28 | 14 |  | 0 | 0 | 0 | 0 |  | - | + | - |
|  | 29 | 14 |  | 0 | 0 | 0 | 0 |  | - | + | - |
|  | 30 | 14 |  | 0 | 0 | 0 | 0 |  | - | - | - |
|  | 31 | 21 |  | 0 | 0 | 0 | 0 |  | - | - | - |
|  | 32 | 21 |  | 2 | 0 | 0 | 2 |  | - | - | - |
| **Vaccinated** | 33 | 3 |  | 0 | 0 | 0 | 0 |  | - | - | - |
|  | 34 | 3 |  | 0 | 0 | 0 | 0 |  | - | - | - |
|  | 35 | 3 |  | 0 | 0 | 0 | 0 |  | - | - | - |
|  | 36 | 3 |  | 0 | 0 | 0 | 0 |  | - | - | - |
|  | 37 | 7 |  | 0 | 0 | 0 | 0 |  | - | - | - |
|  | 38 | 7 |  | 0 | 0 | 0 | 0 |  | - | - | - |
|  | 39 | 7 |  | 0 | 0 | 0 | 0 |  | - | - | - |
|  | 40 | 7 |  | 0 | 0 | 0 | 0 |  | - | - | - |
|  | 41 | 14 |  | 0 | 0 | 0 | 0 |  | - | - | - |
|  | 42 | 14 |  | 0 | 0 | 0 | 0 | - | - | - | - |
|  | 43 | 14 |  | 0 | 0 | 0 | 0 |  | - | + | - |
|  | 44 ^b^ | 10 |  | 0 | 0 | 0 | 0 |  | - | - | - |
|  | 45 | 14 |  | 0 | 0 | 0 | 0 |  | - | + | - |
|  | 46 | 21 |  | 0 | 0 | 0 | 0 |  | - | - | - |
|  | 47 | 21 |  | 0 | 0 | 0 | 0 |  | - | - | - |
|  | 48 | 21 |  | 0 | 0 | 0 | 0 |  | - | - | - |
| **Vaccinated and challenged** | 49 | 3 |  | 1 | 0 | 1 | 0 |  | - | - | - |
|  | 50 | 3 |  | 1 | 1 | 0 | 0 |  | - | - | - |
|  | 51 | 3 |  | 2 | 1 | 0 | 2 |  | + | + | - |
|  | 52 | 3 |  | 1 | 1 | 1 | 2 |  | + | + | - |
|  | 53 | 7 |  | 0 | 1 | 0 | 0 |  | - | - | - |
|  | 54 | 7 |  | 1 | 1 | 0 | 1 |  | - | - | - |
|  | 55 | 7 |  | 1 | 0 | 0 | 1 |  | - | - | - |
|  | 56 | 7 |  | 2 | 1 | 2 | 2 |  | + | - | - |
|  | 57 | 14 |  | 0 | 0 | 0 | 0 |  | - | - | - |
|  | 58 | 14 |  | 0 | 0 | 0 | 0 |  | - | - | - |
|  | 59 | 14 |  | 2 | 0 | 0 | 2 |  | + | + | + |
|  | 60 | 14 |  | 0 | 0 | 0 | 0 |  | - | - | - |
|  | 61 | 21 |  | 0 | 0 | 0 | 0 |  | - | - | - |
|  | 62 | 21 |  | 0 | 0 | 0 | 0 |  | - | - | - |
|  | 63 | 21 |  | 0 | 0 | 0 | 0 |  | - | - | - |
|  | 64 **^a^** | 10 |  | 2 | 2 | 2 | 2 |  | - | + | - |

^a^ bird that died or had to be euthanized due to sever clinical signs

^b^ bird that had to be euthanized due to cannibalism

# Supplementary Figures


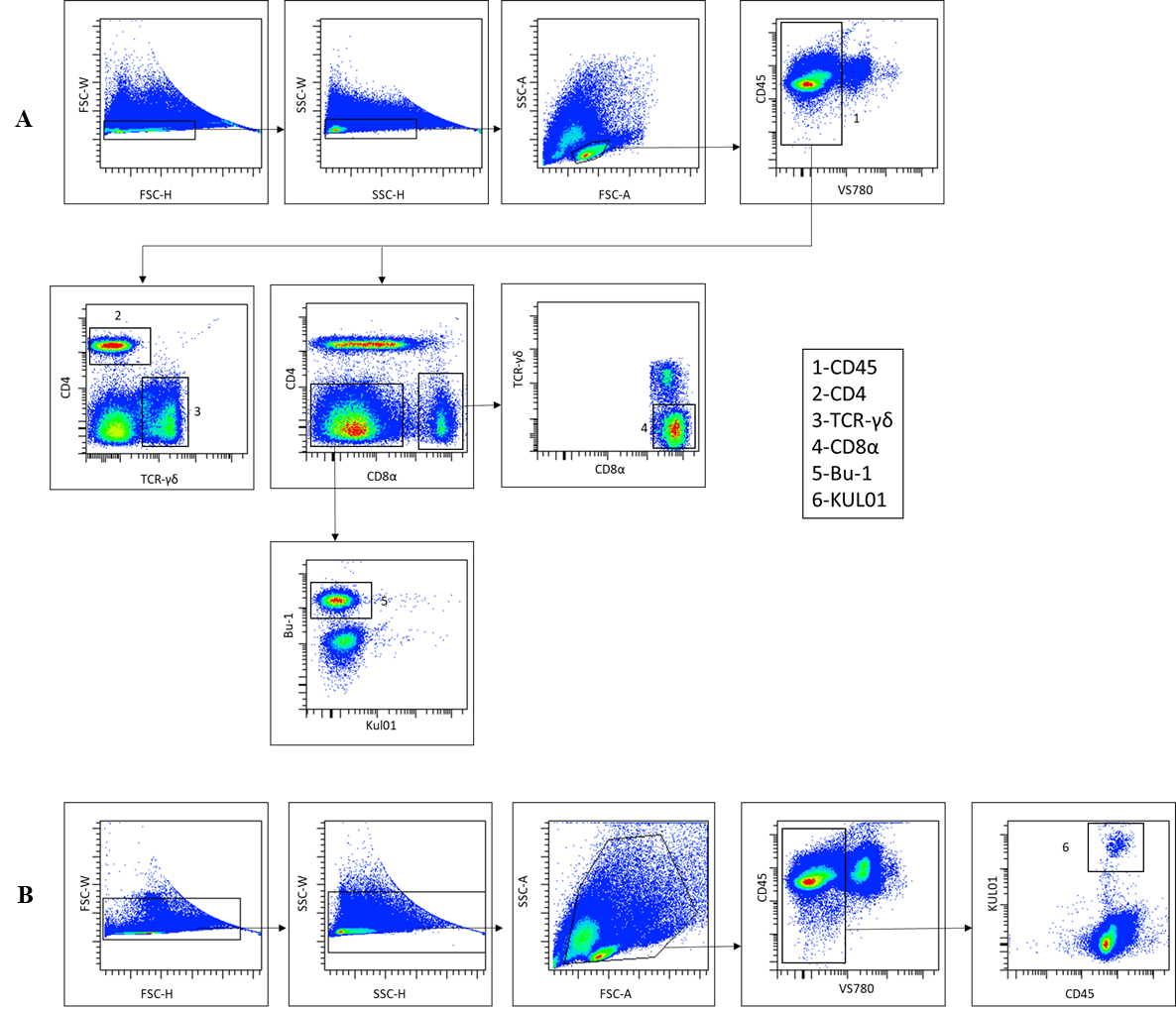


**Supplementary Figure 1.** Gating strategy for immunophenotyping. Gating strategy for lymphocytes and monocyte/macrophages from spleen and lung in multicolor flow cytometry. (A) To identify lymphocytes, FSC-H/FSC-W and SSC-H/SSC-W doublet discrimination gates were applied. Lymphocytes were then selected within a FSC-A/SSC-A plot followed by a dead cell exclusion gate using the BD Horizon^TM^ Fixable Viability Stain 780. Frequencies of cells were determined within total CD45^+^CD4^+^, CD45^+^CD8α^+^(within CD8α^+^ TCR-γδ^-^ cells), total CD45^+^TCR-γδ^+^ as well as CD45^+^Bu-1^+^ (within CD4^-^CD8α^-^ cells) subgates. (B) To identify monocytes/macrophages, an extended FSC-H/FSC-W and SSC-H/SSC-W doublet discrimination gates followed by a FSC-A/SSC-A gates were applied. Dead cells were excluded by using the BD Horizon^TM^ Fixable Viability Stain 780. Frequencies of cells were determined within total CD45^+^ Kul-01^+^ subgates.

**
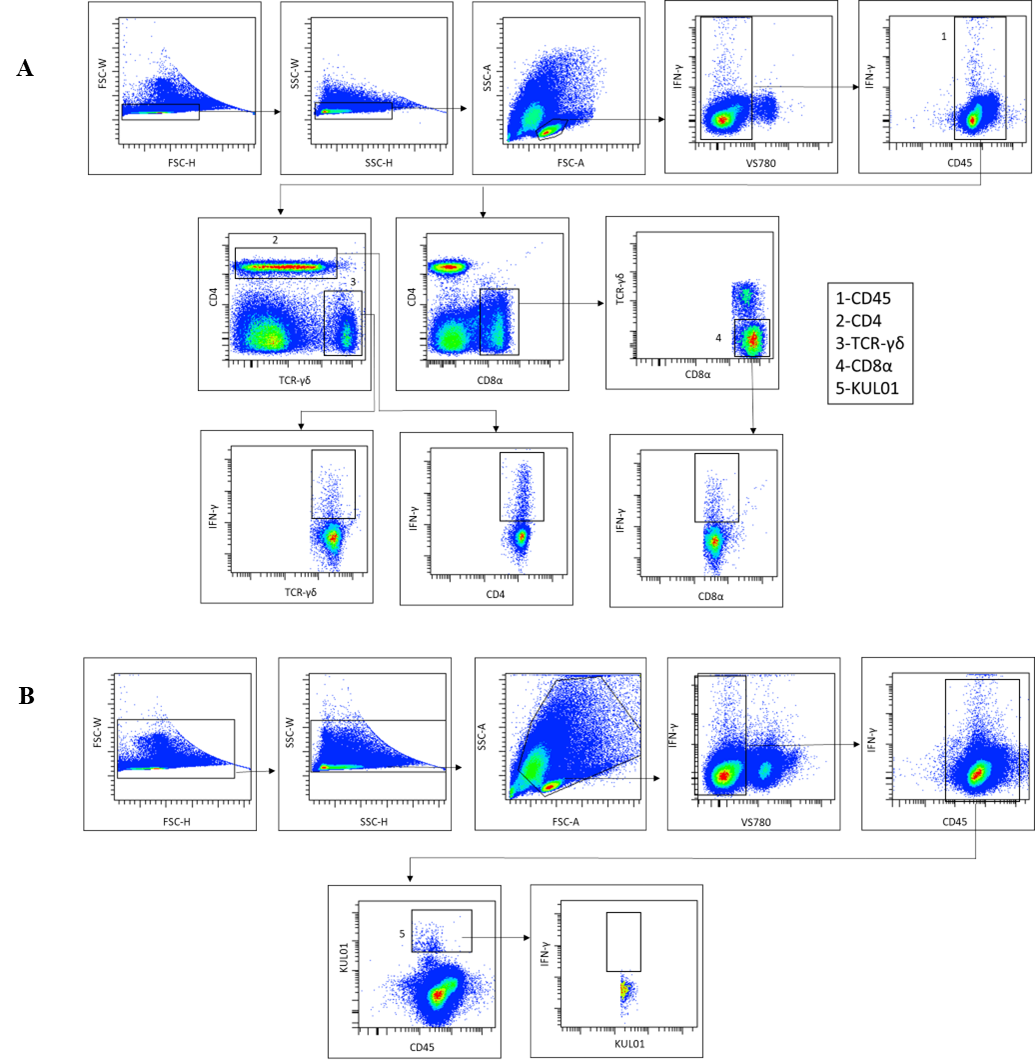
**

**Supplementary Figure 2.** Gating strategy for intracellular cytokine staining. Gating strategy for lymphocytes and monocyte/macrophages from spleen and lung in multicolor flow cytometry. (A) To identify lymphocytes, FSC-H/FSC-W and SSC-H/SSC-W doublet discrimination gates were applied. Lymphocytes were then selected within a FSC-A/SSC-A plot followed by a dead cell exclusion gate using the BD Horizon^TM^ Fixable Viability Stain 780. Frequencies of IFN-γ^+^ cells were determined within total CD45^+^CD4^+^, CD45^+^CD8α^+^(within CD8α^+^ TCR-γδ^-^ cells) and total CD45^+^TCR-γδ^+^ subgates. (B) To identify monocytes/macrophages, an extended FSC-H/FSC-W and SSC-H/SSC-W doublet discrimination gates followed by a FSC-A/SSC-A gates were applied. Dead cells were excluded by using the BD Horizon^TM^ Fixable Viability Stain 780. Frequencies of IFN-γ^+^ cells were determined within total CD45^+^Kul-01^+^ subgates.


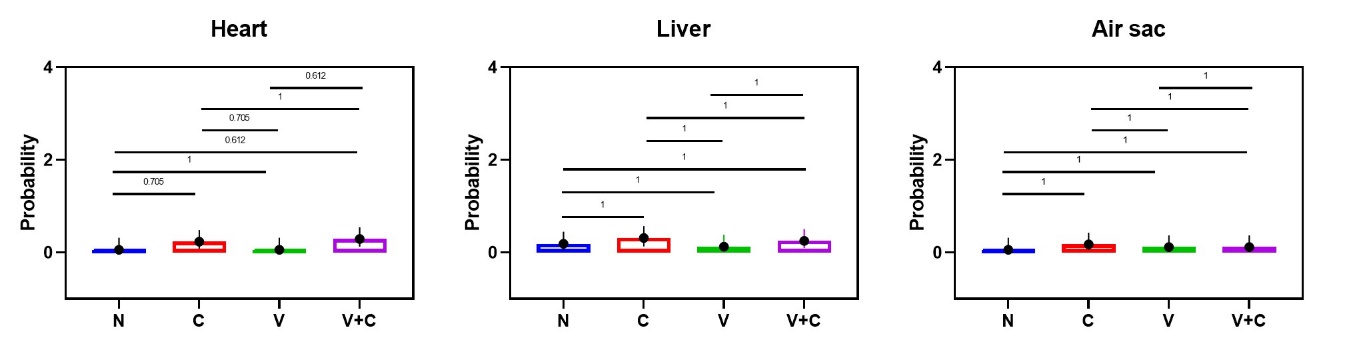


**Supplementary Figure 3.** Predicted probability of bacterial isolation in liver, heart and air sac following vaccination and/or challenge of birds. Comparison of probability of bacterial isolation between experimental groups in liver, heart and air sac.

**
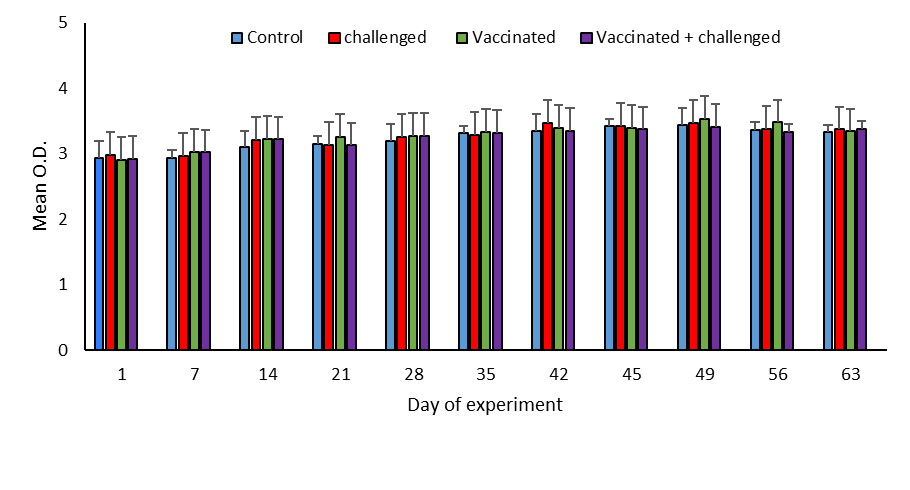
**

**Supplementary Figure 4.** Quantification of IgY antibodies against APEC in sera from birds at different time points before and after challenge.
